# An Engineered Probiotic Consortium Based on Quorum‐Sensing for Colorectal Cancer Immunotherapy

**DOI:** 10.1002/advs.202512744

**Published:** 2025-09-26

**Authors:** Yufei Guo, Mengxue Gao, Lina Wang, Haibin Yuan, Jianan Yin, Jiahao Wu, Xinran Gao, Zhixin Zhu, Yan Zhang, Zichen Wang, He Huang, Guangbo Kang

**Affiliations:** ^1^ School of Synthetic Biology and Biomanufacturing State Key Laboratory of Synthetic Biology Tianjin Key Laboratory of Biological and Pharmaceutical Engineering Tianjin University Tianjin 300072 China

**Keywords:** colorectal cancer, engineered probiotic, immunotherapy, microbial consortium, quorum sensing

## Abstract

Recent progress in synthetic biology has empowered engineered probiotics to sense tumor‐specific physicochemical signals, thereby facilitating targeted in situ drug delivery. Here, an engineered probiotic consortium capable of integrating multiple tumor microenvironment (TME) signals and orchestrating multi‐therapeutic payloads release through an orthogonal quorum‐sensing system is designed. The probiotic consortium can respond to three characteristic TME parameters, pH, hypoxia, and high‐lactate levels, in order to achieve controlled release of lactate depletion enzyme (LdhA) for metabolic environment improvement and the programmed death ligand 1 (PD‐L1) nanobody for immune checkpoint inhibition. Using the humanized PD‐1 mouse model bearing hPD‐L1 MC38 tumor and the humanized peripheral blood mononuclear cells (PBMC) mouse model bearing HT‐29 tumor, it is demonstrated that this self‐regulating microbial consortium achieves sustained oscillations and significantly suppresses tumor progression. Mechanistic studies reveal that the antitumor efficacy activates CD8^+^, CD4^+^, and IFN‐γ^+^ T cells, coupled with diminished immunosuppressive Foxp3^+^ regulatory T cell infiltration. This work advances the development of engineered live biotherapeutic products for cancer therapy and provides a modular platform for microbial consortium‐based precision medicine.

## Graphical abstract

This work develops an engineered probiotic consortium sensing pH, hypoxia, and lactate in the tumor microenvironment. It releases a lactate‐depleting enzyme (LdhA) and a PD‐L1 nanobody. In humanized mouse models (MC38 and HT‐29 tumors), it achieves durable oscillations, suppresses tumors, activates CD8^+^, CD4^+^, IFN‐γ^+^ T cells, and reduces Foxp3^+^ regulatory T cells, highlighting that engineered microbial consortia can be potential therapies.

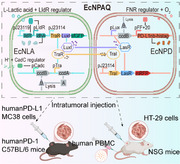

## 1 Introduction

Colorectal cancer (CRC) is the third most common cancer worldwide, accounting for 9.6% of all cancer cases, and it is the second leading cause of cancer‐related deaths, with ≈1.9 million new diagnoses and 900 000 deaths each year.^[^ ^1^ ^]^ In recent years, cancer treatment has seen significant progress with the development of immunotherapy. Inhibitors of the PD‐1/PD‐L1 pathway have been shown to promote tumor regression and enhance survival outcomes in clinical use or under development. These include monoclonal antibodies such as pembrolizumab and nivolumab, which target the programmed death 1 (PD‐1) pathway, and atezolizumab and BMS‐936559, which target the programmed death ligand 1 (PD‐L1) pathway.^[^ ^2^ ^]^

However, many CRC patients remain resistant to immunotherapy.^[^ ^3^ ^]^ The response rates to PD‐1/PD‐L1 blockade vary from 13% to 69%, depending on tumor type.^[^ ^4^, ^5^ ^]^ In order to enhance the efficacy of immune checkpoint inhibitors (ICIs) and improve patient responses, researchers have proposed improved strategies targeting three reasons for resistance. 1) Conventional full‐length anti‐PD‐L1 antibodies often have limited tumor penetration, which undermines their overall effectiveness.^[^ ^6^ ^]^ Anti‐PD‐L1 nanobody (anti‐PD‐L1nb) exhibits excellent tissue penetration, low human immunogenicity, and high affinity, showing great potential in CRC treatment.^[^ ^7^, ^8^, ^9^ ^]^ 2) Engineered bacteria therapies can deliver ICIs directly to the tumor site, overcoming the tissue penetration barriers of antibodies and significantly delaying tumor growth, indicating advantages over traditional antibody therapies.^[^ ^10^, ^11^, ^12^, ^13^ ^]^ Although biosafety needs to be continuously evaluated, production costs are lower than those of CAR‐T cell therapy. 3) The high‐lactate TME induces the activation of PD‐L1 in tumor cells, thereby contributing to tumor cells' protection from targeting by cytotoxic T‐cells.^[^ ^14^, ^15^ ^]^ And lactate enhances lactylation of apolipoprotein C‐II protein, thereby leading to regulatory T cell accumulation and immunotherapy resistance.^[^ ^16^ ^]^ Therefore, researchers employ lactate oxidase or lactate dehydrogenase to effectively reduce TME lactate levels.^[^ ^17^ ^]^

Previous studies have utilized single‐engineered bacteria to deliver anti‐PD‐L1nb or lactate‐degrading proteins in situ.^[^ ^18^, ^19^, ^20^ ^]^ However, these studies are deficient in precise regulatory mechanisms and exhibit unsatisfactory in vivo safety. Researchers further constructed synchronized lysis circuits with a quorum‐sensing system (QS), in which engineered bacteria could detect the number of population or a specific environmental signal.^[^ ^21^, ^22^ ^]^ However, these single engineered bacteria are subject to regulation by one or two signals. In addition, crosstalk of natural QS is still confronted with the toxic effects of engineered bacterial leakage in vivo. In order to respond to more signals and deliver multiple therapeutics, we decided to construct an engineered probiotic consortium regulated by an orthogonal QS system.

Furthermore, to enhance biosafety, we tried to select an intestinal probiotic as a delivery system for live biotherapeutic products targeting tumors.^[^ ^23^, ^24^, ^25^ ^]^ *Escherichia coli* Nissle 1917 (EcN) is considered a strong candidate due to its unique ability to infiltrate and colonize tumors, especially in the hypoxic core.^[^ ^26^ ^]^ And it does not colonize other organs, making it an excellent choice for biosafety.^[^ ^27^, ^28^ ^]^ On one hand, EcN can improve gut microbiota, thereby modulating the efficacy of PD‐1 pathway inhibitors in cancer.^[^ ^29^, ^30^, ^31^, ^32^ ^]^ On the other hand, EcN has been shown to promote apoptosis in HT‐29 and CT26 cells by regulating key signaling pathways, such as Bax/Bcl‐xL and AKT/PTEN.^[^ ^33^ ^]^ Thus, we have chosen EcN as a delivery system for tumor‐specific localized release.

**Figure 1:**
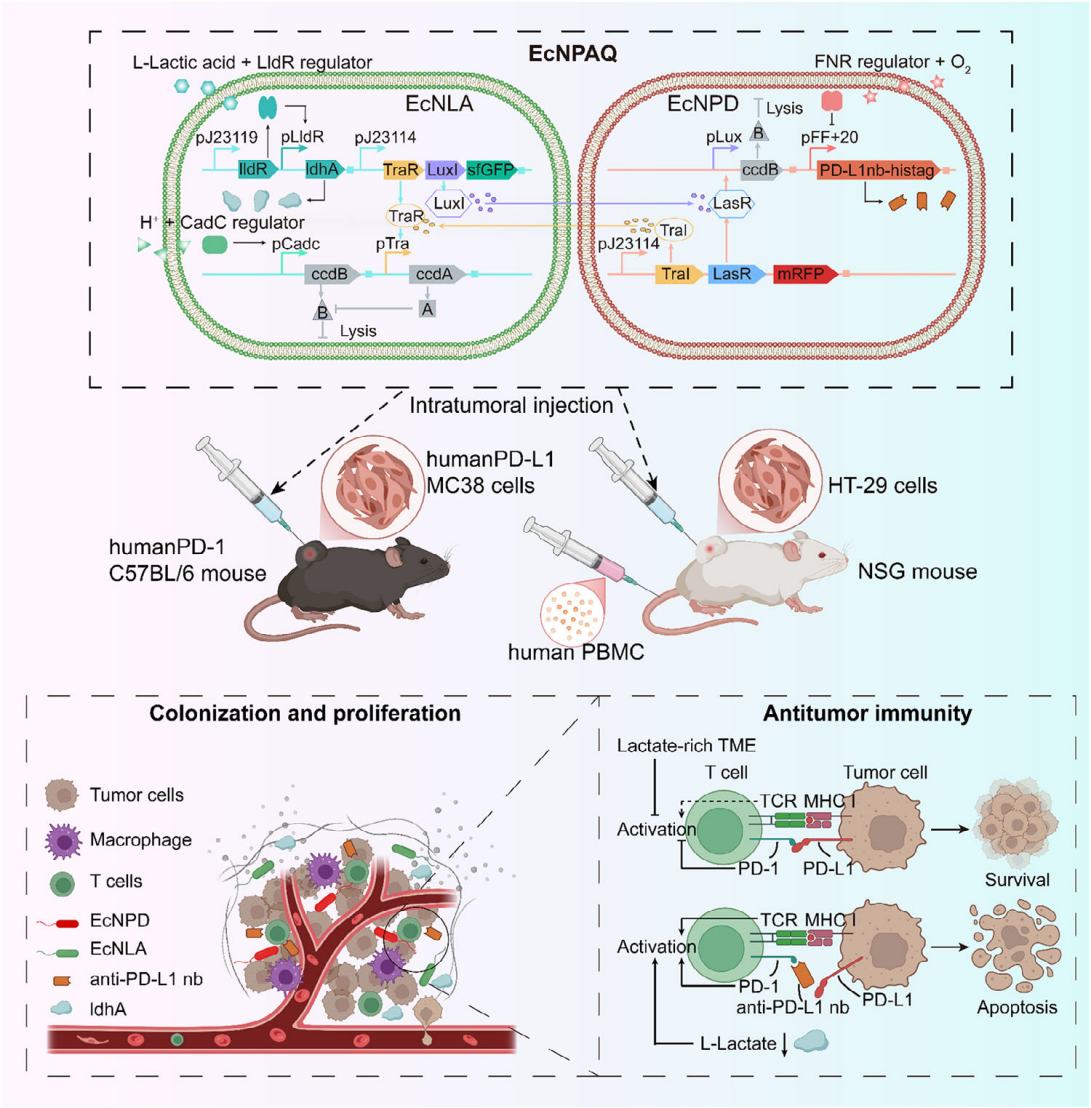
Schematic diagram of the components and sensory pathways of EcNPAQ for diagnosis and treatment of CRC.

In summary, we have developed a mixed system, EcNPAQ, composed of two interacting EcN strains, forming an engineered probiotic consortium that responds to acidic, hypoxic, and high‐lactate TME. EcNLA expresses lactate dehydrogenase for metabolic improvement of the TME, while EcNPD delivers anti‐PD‐L1nb. Cross‐regulation through the LasR‐plux and TraR‐ptra QS systems can prevent signal interference while achieving self‐sustaining population dynamics. Computational simulations optimized the cultivation ratio of the mixed system to ensure sustained and balanced release of therapeutic agents. To evaluate treatment efficacy, we established two mouse models, hPD‐1 mouse model bearing hPD‐L1 MC38 tumor and humanized peripheral blood mononuclear cells (PBMC) mouse model bearing human colon tumor HT‐29, to validated the effects of intratumoral injection of EcNPAQ (**Figure** **1**). This study demonstrates a responsive engineered probiotic consortium that achieves oscillating and durable release of therapeutic molecules, presenting a safe and controllable approach with enormous potential in immunotherapy for gastrointestinal tumors.

The engineered probiotic consortium (EcNPAQ) sense and detect pH, L‐lactate and oxygen levels. And EcNPAQ express orthogonal quorum‐sensing signals to secrete the therapeutic molecules within the TME. Moreover, EcNPAQ produces real‐time signals (fluorescence) and therapeutic molecules (lactate dehydrogenase and anti‐PD‐L1nb) to treat hPD‐L1 MC38 tumor in a humanized PD‐1 mouse model and human HT‐29 tumor in a humanized PBMC mouse model. Created with Biorender.com.

## 2 Design and Characterization of Engineered Parts and Modules

**Figure 2:**
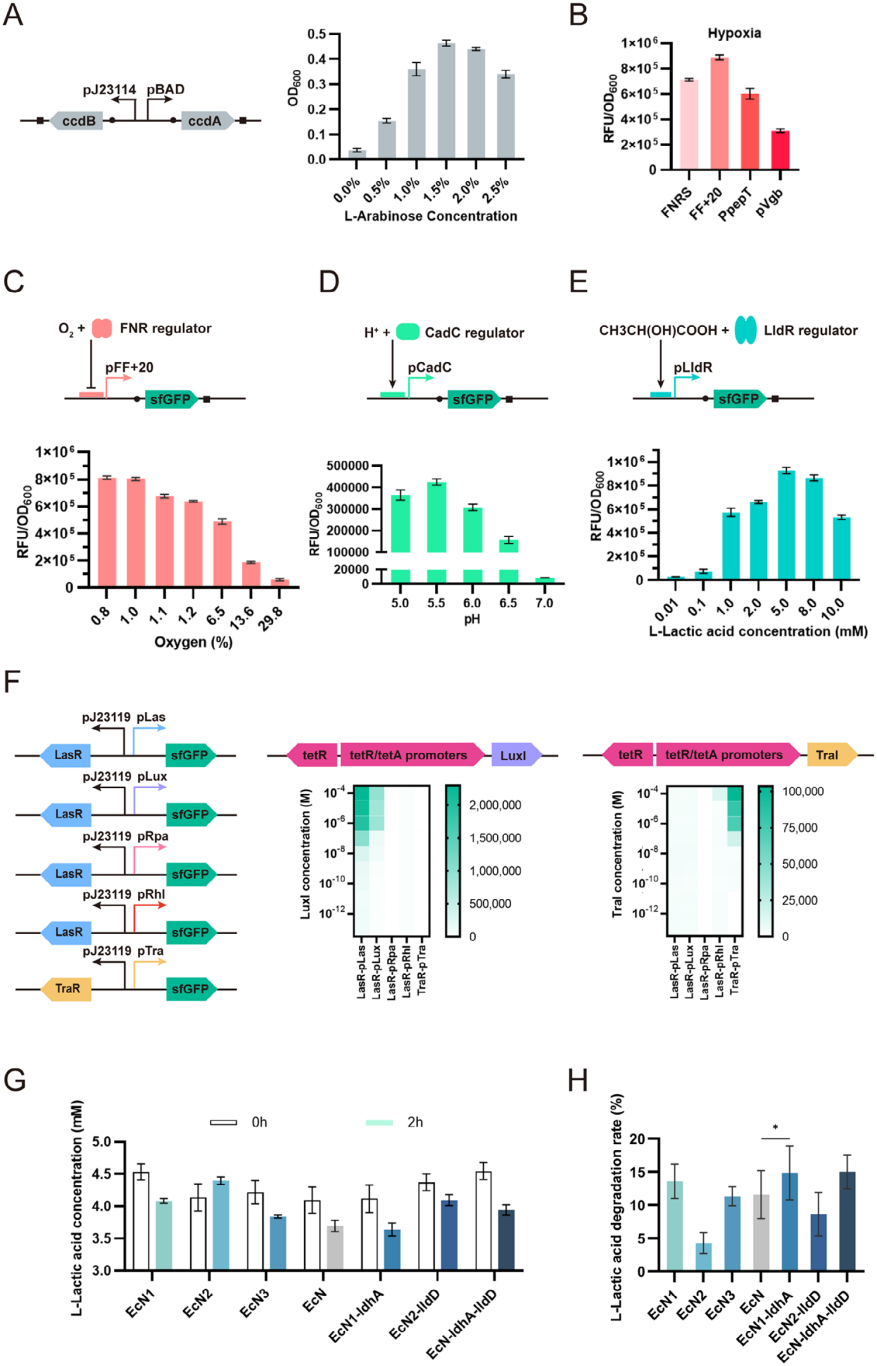
Design and characterization of hypoxia, lactate and pH biosensors. A) The bacterial toxin‐antitoxin systems, ccdA antitoxin protein is expressed from an arabinose‐inducible pBAD promoter and ccdB toxin protein is expressed from a constitutive promoter. Strains with low L‐arabinose concentrations showed the prominent lysis rate of ccdB protein. B) The hypoxia biosensor uses the pFNRS, pFF+20, pPepT, and pVgb promoters that relies on dimerized FNR to drive gene expression under low oxygen levels. The pFF+20 promoter has the strongest mean fluorescence in response to hypoxic conditions. The mean fluorescence was calculated as the ratio of fluorescence/OD_600_ (mean ± SEM, *n* = 3 biologically independent experiments). C) The pFF+20 promoter is regulated by dimerized transcriptional factor FNR. As oxygen concentrations increased, mean fluorescence decreased gradually. D) The pH biosensor, which is based on the pCadC system, is regulated by the membrane‐tethered transcriptional factor CadC. As pH levels decreased, mean fluorescence first increased and then decreased. E) The lactate biosensor contains constitutive production of a LldR repressor, which derepresses and activates the pLldR promoter in the presence of lactate. As L‐lactate concentrations increased, mean fluorescence first increased and then decreased. F) Validation of different orthogonal QS systems. To prevent high leakiness and achieve high expression, the LuxI‐LasR‐plux and TraI‐TraR‐ptra systems were selected at final. G) Validation of different systems consisting of two types of lactate dehydrogenase. The strain overexpressed ldhA genes exhibited the most prominent L‐lactic acid degradation. H) The strain overexpressed ldhA genes exhibited the highest L‐lactic acid degradation rate. Data in A–H) are presented as means ± s.e.m. *n* = 3. Each data point represents the mean of three technical replicates. p values were calculated by one‐way analysis of variance (ANOVA) with Tukey's post‐test. *p < 0.05.

For controlled bacterial lysis, we repurposed the ccdAB toxin‐antitoxin system from the *E. coli* F plasmid. The DNA gyrase inhibitor ccdB induces double‐strand breaks, while its antidote ccdA neutralizes toxicity through complex formation.^[^ ^34^ ^]^ The plasmid is of a dual‐expression plasmid, comprising two components: ccdA, with an arabinose‐inducible pBAD promoter, and ccdB, with a J23114 constitutive promoter. The presence of these elements enables tunable lysis in EcN. Dose‐dependent arabinose supplementation revealed complete growth inhibition below 0.5% arabinose, whereas concentrations ≥1.5% maintained viability. It confirmed ccdA‐mediated protection (**Figure** **2**A).

To develop programmable bacterial biosensors for TME discrimination, we focused on three pathological signals: hypoxia, acidic pH, and high‐lactate levels. Each rigorously validated through cross‐species quantification in murine and human tumors.^[^ ^35^, ^36^, ^37^, ^38^ ^]^ Our modular biosensor design integrates condition‐specific promoters with a superfolderGFP (sfGFP) reporter system, enabling real‐time environmental sensing.

Hypoxia sensing promoters regulated by the oxygen‐responsive transcription factor FNR (fumarate and nitrate reduction regulator) in EcN. Under hypoxia, FNR forms a transcriptionally active homodimer via a [4Fe‐4S]^2^⁺ cluster. Oxygen exposure triggers cluster disassembly and monomerization, leading to the termination of promoter activity.^[^ ^39^ ^]^ Systematic screening of four FNR‐dependent promoters identified pFF+20 as optimal (Figure 2B), demonstrating hypoxia‐inducible sfGFP expression with inverse correlation to oxygen concentration (Figure 2C).

Transcriptional regulator CadC is an acid‐sensing protein.^[^ ^40^ ^]^ pH detection was achieved using the CadC‐regulated pCadC promoter. This membrane‐tethered pH sensor exhibited increased activity in acidic environments compared to neutral pH.^[^ ^41^ ^]^ Activation of the biosensor was observed within physiologically relevant tumor pH ranges (Figure 2D).

Lactate quantification leveraged the lldPRD operon's native regulation. Our plasmid‐based system combined a lactate‐responsive promoter (pLldR) driving sfGFP with constitutive expression of the lactate‐binding repressor LldR.^[^ ^42^ ^]^ Maximum induction occurred at 5 mM L‐lactate (Figure 2E), beyond which sensor output declined due to growth inhibition.

Next, to achieve a dual functionality of degrading lactic acid and releasing anti‐PD‐L1nb simultaneously, we aimed to create an engineered probiotic consortium comprising two engineered EcN strains with complementary functions. This necessitated the implementation of two orthogonal QS systems to prevent signal crosstalk and promoter crosstalk in common QS systems. The Tra and Las systems, although promoter‐orthogonal, exhibit potential signal crosstalk. While the LasR‐plas system demonstrates higher fluorescence response, it also exhibits greater leakage.^[^ ^43^ ^]^ Thus, we selected other components employing distinct QS systems: the TraI/TraR system from *Agrobacterium tumefaciens* (ligand 3OC8‐HSL as an autoinducer), the LasI/LasR system from *Pseudomonas aeruginosa* (ligand 3OC12‐HSL), the LuxI/LuxR system from *Vibrio fischeri* (ligand 3OC6‐HSL), and the RpaI/RpaR system from *Rhodopseudomonas palustris* (ligand pC‐HSL). To evaluate signal orthogonality, we performed preliminary tests using varying concentrations of 3OC6HSL, 3OC8‐HSL, and 3OC12HSL. The LasR‐plux and TraR‐ptra systems exhibited optimal signal orthogonality within a concentration range (10−100 000 nM for 3OC6HSL and 3OC8HSL). Due to high leakage, 3OC12HSL was deemed unsuitable for constructing orthogonal systems. Additionally, we assessed whether self‐produced autoinducers could achieve orthogonality. The LasR‐plux and TraR‐ptra systems continued to exhibit the most effective orthogonality among the five systems evaluated. Consequently, we selected the LasR‐plux and TraR‐ptra systems for our engineered probiotic consortium system (Figure 2F).

Lastly, we investigated two distinct enzymatic systems. LdhA, an NAD⁺‐dependent lactate dehydrogenase catalyzing pyruvate formation coupled with NAD⁺ reduction to NADH. And LldD, a cytochrome c‐linked dehydrogenase mediating lactate oxidation through ferricytochrome c reduction. To establish an optimized lactate‐consuming chassis, we engineered a series of strains through chromosomal deletions and plasmid‐based complementation (Table S1, Supporting Information). Specifically, EcN1 (Δ*lldD*) and EcN2 (Δ*ldhA*) harbored single‐gene knockouts, while EcN3 (Δ*lldD*Δ*ldhA*) represented a double‐deletion mutant. Genetic complementation generated EcN1‐ldhA (Δ*lldD*/*ldhA*+), EcN2‐lldD (Δ*ldhA*/*lldD*+), and EcN‐ldhA‐lldD (*ldhA*+/*lldD*+) strains. Following 2 h cultivation in 5 mM lactic acid, the EcN1‐ldhA strain demonstrated the most efficient lactate clearance, reducing extracellular lactate by 22.37% (Figure 2G,H). This superior performance likely stems from eliminating competing LldD‐mediated electron flux. Based on these findings, we selected the EcN1‐ldhA chassis with augmented LdhA expression as the platform for subsequent applications requiring enhanced lactate assimilation capacity.

## 3 Design of an Intelligent Engineered Probiotic Consortium for the Treatment of CRC

We have integrated the aforementioned environmental signal sensing module, therapeutic molecule production module, and population behavior regulation module (QS system) to create an intelligent engineered probiotic consortium, designated EcNPAQ (EcN with anti‐PD‐L1nb, lactate dehydrogenase and QS system), for the treatment of CRC.^[^ ^44^ ^]^ EcNPAQ is engineered to colonize tumors while effectively sensing the acidic, hypoxic, and high‐lactate TME of CRC. The consortium consists of two key components: EcNLA (EcN with lactate dehydrogenase), which degrades lactic acid at appropriate times, and EcNPD (EcN with anti‐PD‐L1nb), which heals by periodically accumulating and releasing anti‐PD‐L1nb. Furthermore, these two strains interact dynamically, maintaining a balanced ecosystem.

**Figure 3:**
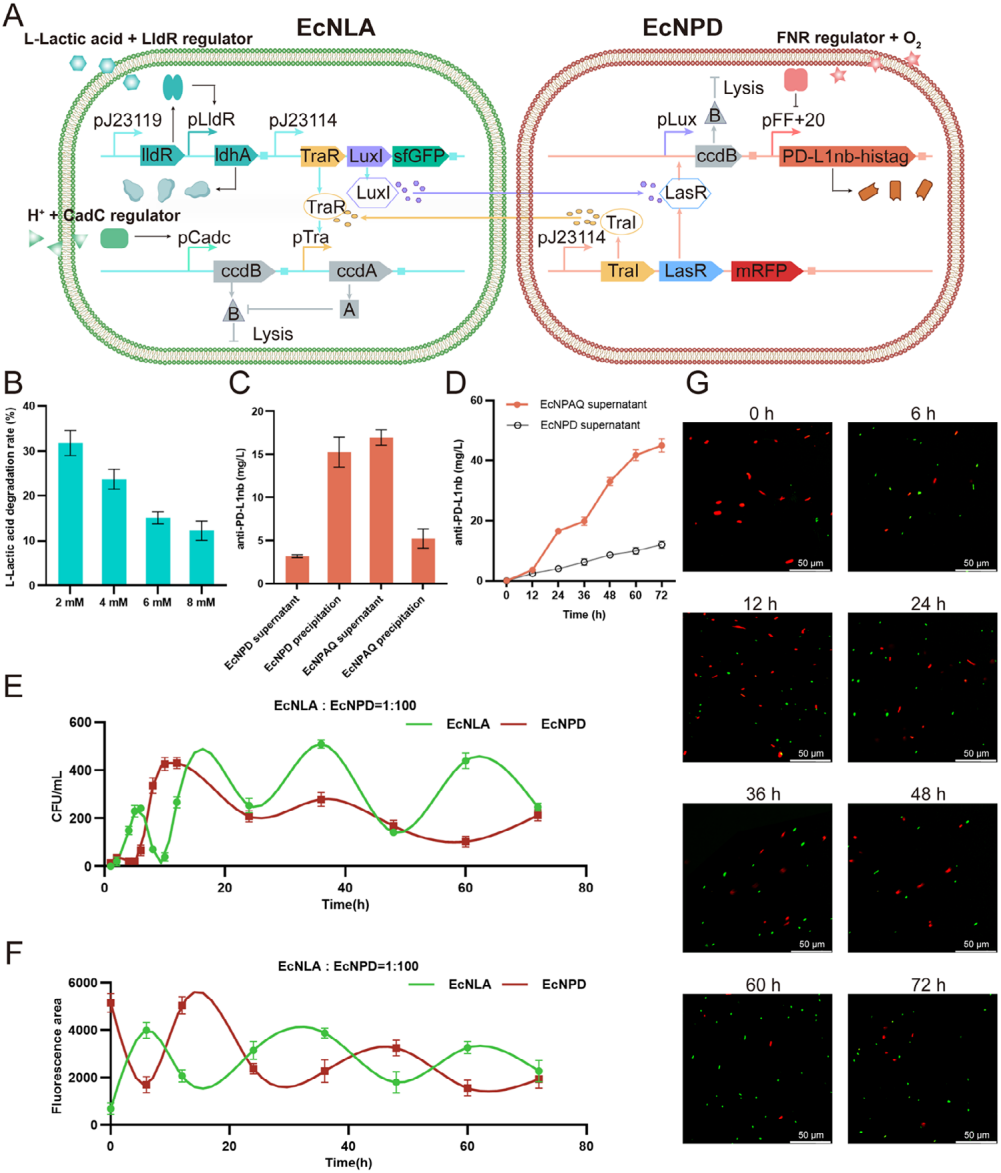
Design and functional characterization of the intelligent engineered probiotic consortium. A) Schematic diagram of the genetic circuit and sensory pathways of the engineered probiotic consortium EcNPAQ. B) As L‐lactate concentrations increased (2, 4, 6, and 8 mM), the L‐lactic acid degradation rate of EcNPAQ supernatant decreased. C) Supernatants and precipitates of EcNPD and EcNPAQ were sampled after 24 h of incubation, respectively. EcNPAQ was able to accumulate and release more anti‐PD‐L1nb into the environment. D) Quantification of the secretion level of anti‐PD‐L1nb by ELISA. The EcNPAQ was cultured in 1 L fermenters and sampled twice a day over 3 days. Subsequently, the anti‐PD‐L1nb concentration was measured in supernatant LB medium by ELISA. Lysis events correspond to peaks of anti‐PD‐L1nb concentration. E) The EcNPAQ was cultured in 1 L fermenters and sampled over 3 day. Samples were plated on LB agar plates and counted for c.f.u. of green (EcNLA) and red (EcNPD) fluorescence. The engineered probiotic consortium established an oscillatory equilibrium. F) The calculation of fluorescence area in images by confocal fluorescence microscopy. G) The dynamic equilibrium of green (EcNLA) and red (EcNPD) fluorescence at different times was visualized by confocal fluorescence microscopy. Scale bar, 50 µm. Data in (B–D) are presented as means ± s.e.m., *n* = 3 independent experiments. Each data point represents the mean of three technical replicates.

To regulate the dynamic equilibrium among the two microbial populations, we drew inspiration from the predator‐prey synthetic gene circuit system, which simulates natural population oscillations and achieves density homeostasis through two QS modules (LuxI/LuxR and LasI/LasR).^[^ ^45^, ^46^ ^]^ Both EcNLA and Ec NPD were transformed with two engineered plasmids (**Figure** **3**A). Additionally, we knocked out the *lldD* gene in the EcNLA genome.

In this system, EcNLA can activate the pLldR promoter under high‐lactate conditions, inducing the expression of the lactate‐depleting enzyme LdhA. However, since LdhA accumulates intracellularly, it can only consume intracellular lactate that enters the cell via lactate transporters MCTs. High lactate concentrations in the TME suppress immune cell function, thereby inhibiting the efficacy of PD‐1/PD‐L1 immunotherapy.^[^ ^47^, ^48^, ^49^ ^]^ As intracellular LdhA cannot effectively degrade lactate, its release is necessary. EcNLA can also activate the pCadC promoter in acidic environments, leading to the expression of ccdB, which triggers EcNLA lysis and releases LdhA into the tumor. This helps reduce lactate levels in the TME and modulates the metabolic environment within the tumor. Concurrently, the density of EcNLA decreases. The reduced EcNLA density results in decreased production of the LuxI signaling molecule, preventing the pLux promoter in EcNPD from expressing ccdB. This allows EcNPD to proliferate continuously. Meanwhile, pFF+20 promoter responds to anaerobic conditions and continuously produces PD‐L1nb. When EcNPD reaches a high density, it produces TraI signaling molecules, which diffuse into EcNLA and bind to TraR. This induces the expression of ccdA in EcNLA, which neutralizes ccdB, preventing further lysis and allowing EcNLA to gradually recover in density. As EcNLA density increases, it produces abundant LuxI signals that diffuse into EcNPD. There, LuxI binds to LasR and activates the pLux promoter, driving expression of ccdB. This triggers extensive lysis of EcNPD, releasing the accumulated PD‐L1nb into the tumor to exert immunotherapeutic effects.

We first evaluated the lactate degradation capacity of EcNPAQ. Given that normal human blood lactate concentrations range from 0.5 to 1.7 mM, and TME typically exhibit lactate levels of 2 to 8 mM, we tested this microbial consortium with lactate concentrations of 2, 4, 6, and 8 mM over a 2 h incubation. The lactate degradation rate decreased with increasing lactate concentration, achieving a maximum rate of 36.92% at 2 mM concentrations (Figure 3B). A comparison of lactate degradation reveals that EcNPAQ achieves its maximum lactate consumption at 4 mM and consumes up to ≈1 mM. Therefore, at concentrations of 4, 6, and 8 mM, a higher initial lactate concentration leads to a lower lactate degradation rate.

Next, we assessed the expression capacity of anti‐PD‐L1nb in EcNPAQ. Centrifuge the EcNPD culture and EcNPAQ culture grown overnight in anaerobic condition, and collect the supernatant and precipitate separately. The precipitate was washed and resuspended in PBS after sonication. Both the supernatant and precipitate were purified using Ni columns, with anti‐PD‐L1nb concentration measured via A280. Although EcNPD can produce and accumulate anti‐PD‐L1nb, nanobody release is hindered without 3OC6‐HSL signaling from EcNLA. Consequently, in EcNPD culture, the supernatant was nearly devoid of antibody, while the precipitate had significant accumulation of it. In contrast, in EcNPAQ culture, anti‐PD‐L1nb was primarily found in the supernatant, indicating effective accumulation and release (Figure 3C).

In experiments simulating anaerobic conditions with low pH and high lactate levels, we discovered that when the initial ratio of EcNLA to EcNPD was 1:1, no oscillatory behavior was observed. Since the “prey” EcNPD strain may require a larger population to sustain the “predator” EcNLA strain, we opted to utilize the modified Lotka‐Volterra equations for our computational simulations.^[^ ^50^, ^51^ ^]^ We observed periodic lysis events characterized by peaks in fluorescent reporter expression, which correspond to population lysis.^[^ ^50^ ^]^ After evaluating initial ratios of 100:1, 10:1, 1:1, 1:10, and 1:100 between EcNLA and EcNPD, we found that the 1:100 ratio was the most effective and stable (Figure S1, Supporting Information). As a result, we chose an initial EcNLA to EcNPD ratio of 1:100 as the experimental condition.

Finally, we tested EcNPAQ's ability to oscillate periodically under a complicated environment, as bacteria sense multiple physiological signals in vivo. We evaluated the functional activity of the biosensors and modules under these conditions, indicating that they can be employed combinatorially. The EcNPAQ was cultured in 1 L fermenters and sampled twice a day over 3 days. The experimental setup involved anaerobic culture at pH 5.5, with a stable lactate concentration of 2 mM. EcNLA was inoculated at a ratio of 1:10000, while EcNPD was inoculated at a ratio of 1:100. Over 72 h, the anti‐PD‐L1nb concentration increased over time (Figure 3D). Both EcNLA and EcNPD demonstrated synchronized oscillations, achieving a cyclic dynamic equilibrium that continuously accumulated and released lactate dehydrogenase and anti‐PD‐L1nb (Figure 3E). We obtained confocal fluorescence images of the EcNPAQ at eight time points. Quantitative analysis of the fluorescent areas in these images revealed a similar dynamic equilibrium (Figure 3F,G). The amplitude of the oscillation gradually decreases, with an approximate frequency of 24 h.

## 4 Biosensors in Physiological Environments and EcNPAQ Therapeutic Effectiveness Measured by Macrophage Phagocytosis

**Figure 4:**
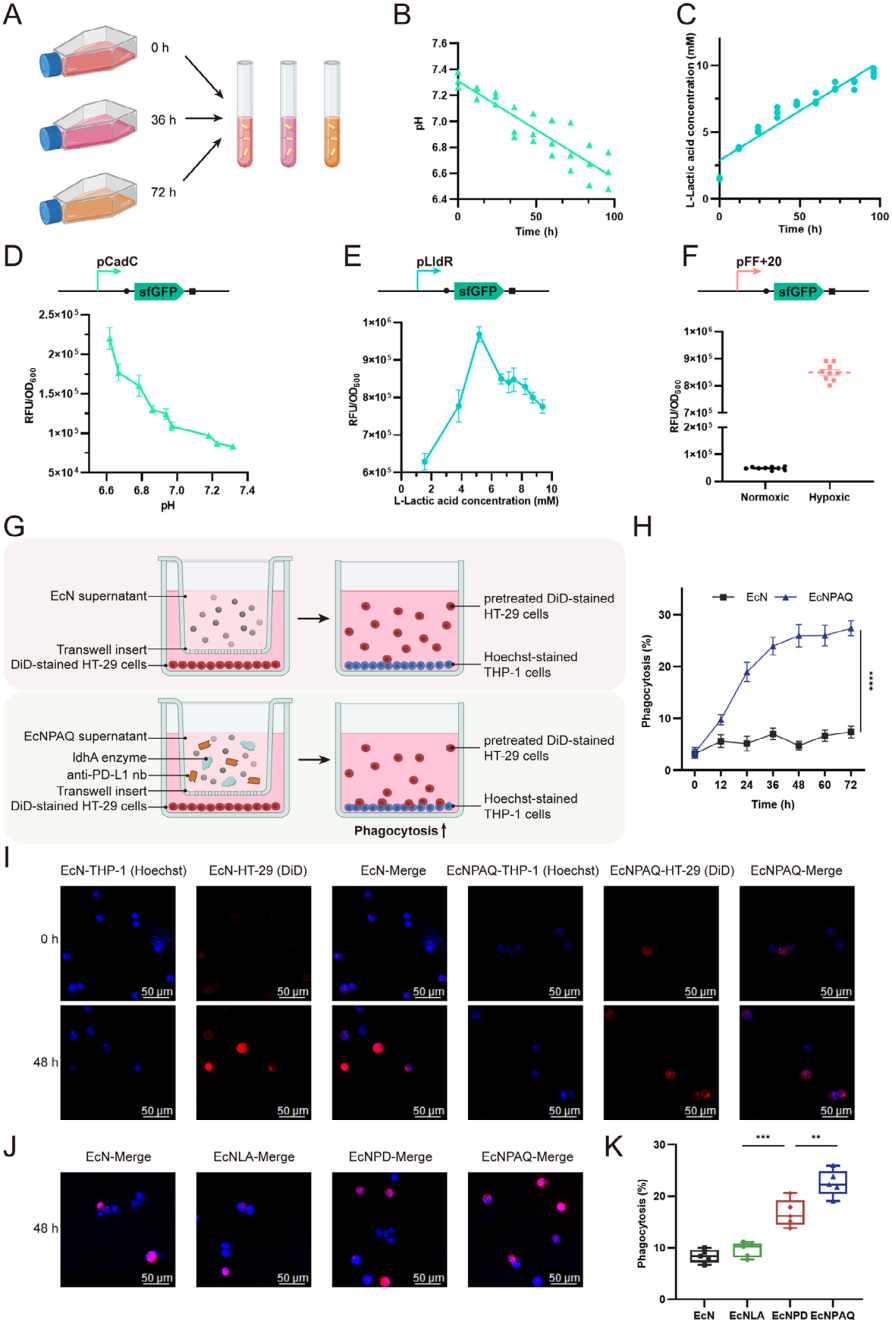
Engineered biosensors respond to physiological signals and phagocytosis to detect EcNPAQ activities. A) Cell culture medium supernatant from HT29 cancer cell lines was collected twice a day over 3 d and then cultured with three bacterial biosensors (pCadC, pLldR, and pPepT). Created with Biorender.com. B) Levels of pH of HT29 cell supernatants over 3 days. The pH level from cell line supernatants showed decreasing trends. C) Levels of L‐lactic acid concentration of HT29 cell supernatants over 3 days. Lactate concentration of the collected supernatants exhibited an increasing trend. D–F) Mean fluorescence activation of pH (B), L‐lactic acid (C), and hypoxia (D) biosensors when cultured in the collected supernatant overnight at 37 °C. The hypoxia biosensor cultured in cell medium supernatant was grown under conditions with or without oxygen. The GFP signal from the hypoxia biosensor was normalized to the constitutive promoter control. G) In vitro phagocytosis of DiD‐labeled HT29 cells that were pretreated with EcN or EcNPAQ supernatant by THP‐1 cells. Created with Biorender.com. H) The ratio of DiD^+^ HT29 cells to total Hoechst 33 342^+^ THP‐1 cells was counted from each confocal microscopy photo to score phagocytosis. I) Microscopic visualization of the phagocytosis of EcN and EcNPAQ pretreated at different co‐culture times. HT29 cells were stained with DiD (red), THP‐1 cells were stained with Hoechst 33 342 (blue). Scale bar, 50 µm. J) Microscopic visualization of the phagocytosis of EcN, EcNLA, EcNPD, and EcNPAQ supernatant at 48 h. Scale bar, 50 µm. K) The phagocytosis rate of EcN, EcNLA, EcNPD, and EcNPAQ supernatant at 48 h. Data in B–E) are presented as means ± s.e.m., *n* = 3. Data in F) are presented as means ± s.e.m., *n* = 9. Data in H) and K) are presented as means ± s.e.m., *n* = 5. *p*‐values were calculated by one‐way ANOVA with Tukey's post‐test. *p < 0.05, **p < 0.01, ***p < 0.001 and ****p < 0.0001.

To characterize bacterial biosensors in vivo, we first assessed their ability to sense the metabolic activity of mammalian cell cultures in vitro (**Figure** **4**A). HT‐29 cells were cultured for four days, and then L‐lactate and pH levels were measured in the harvested medium supernatant (Figure 4B,C). Subsequently, we cultured biosensor‐containing bacteria (pFF+20‐sfGFP, pCadC‐sfGFP, and pLldR‐sfGFP) in the collected supernatant and measured the fluorescence of these strains after 16 h. We observed a significant increase in bacterial fluorescence signals corresponding to higher lactate concentrations and lower pH levels (Figure 4D,E). Then bacteria were incubated overnight in the supernatant under both normoxic and hypoxic conditions, revealing a high fluorescence signal exclusively in the hypoxic environment (Figure 4F). In summary, these results demonstrate that our bacterial biosensors can effectively detect biochemical signatures in physiological environments. Using a Transwell co‐culture system with HT‐29 cells and EcNPAQ, we demonstrated that EcNPAQ can deplete lactate and release PD‐L1nb under physiological environments (Figure S2, Supporting Information).

Prior studies indicate that anti‐PD‐L1 antibody significantly enhance macrophage proliferation and infiltration in vivo, promoting a proinflammatory phenotype.^[^ ^52^ ^]^ In our study, we evaluated the phagocytosis rate of activated macrophages against colon cancer cells. The macrophage and HT‐29 cell co‐culture experiments were conducted in TME‐mimicking conditions as mentioned above. HT29 cells (red) and macrophages (blue) were stained with DiD and Hoechst 33 342 dyes, respectively. After co‐culturing EcN or EcNPAQ supernatants from different time points with HT29 cells, we utilized confocal microscopy to assess macrophage phagocytosis rates (Figure 4G). Over one hundred images were captured, and we analyzed one hundred randomly selected blue‐fluorescent macrophages to quantify those exhibiting red and blue merged fluorescence, indicative of phagocytosis (Figure 4I; Figure S3, Supporting Information). The phagocytosis rates were significantly different between groups, with the EcNPAQ supernatant achieving ≈27.39% phagocytosis, compared to 7.38% in the EcN group (Figure 4H). Notably, the increasing macrophage phagocytosis suggested that anti‐PD‐L1nb enhances macrophage phagocytosis of tumor cells in a combinatorial, dose‐dependent manner. Furthermore, EcNPD was cultured with 10^−6^ M LuxI, while EcNLA was cultured with 10^−6^ M TraI signal. The supernatants of EcNPD and EcNLA were collected after 48 h. The supernatant of EcN and EcNPAQ without signal induction was also collected. Compared to the phagocytosis rate observed in the EcNPAQ group, the EcNPD group releasing only PD‐L1nb showed a lower phagocytosis rate, and the EcNLA group releasing only LdhA did not increase the phagocytosis rate (Figure 4J,K).

## 5 Antitumor Efficacy of EcNPAQ against MC38 Tumors in a Humanized PD‐1 Mouse Model

**Figure 5:**
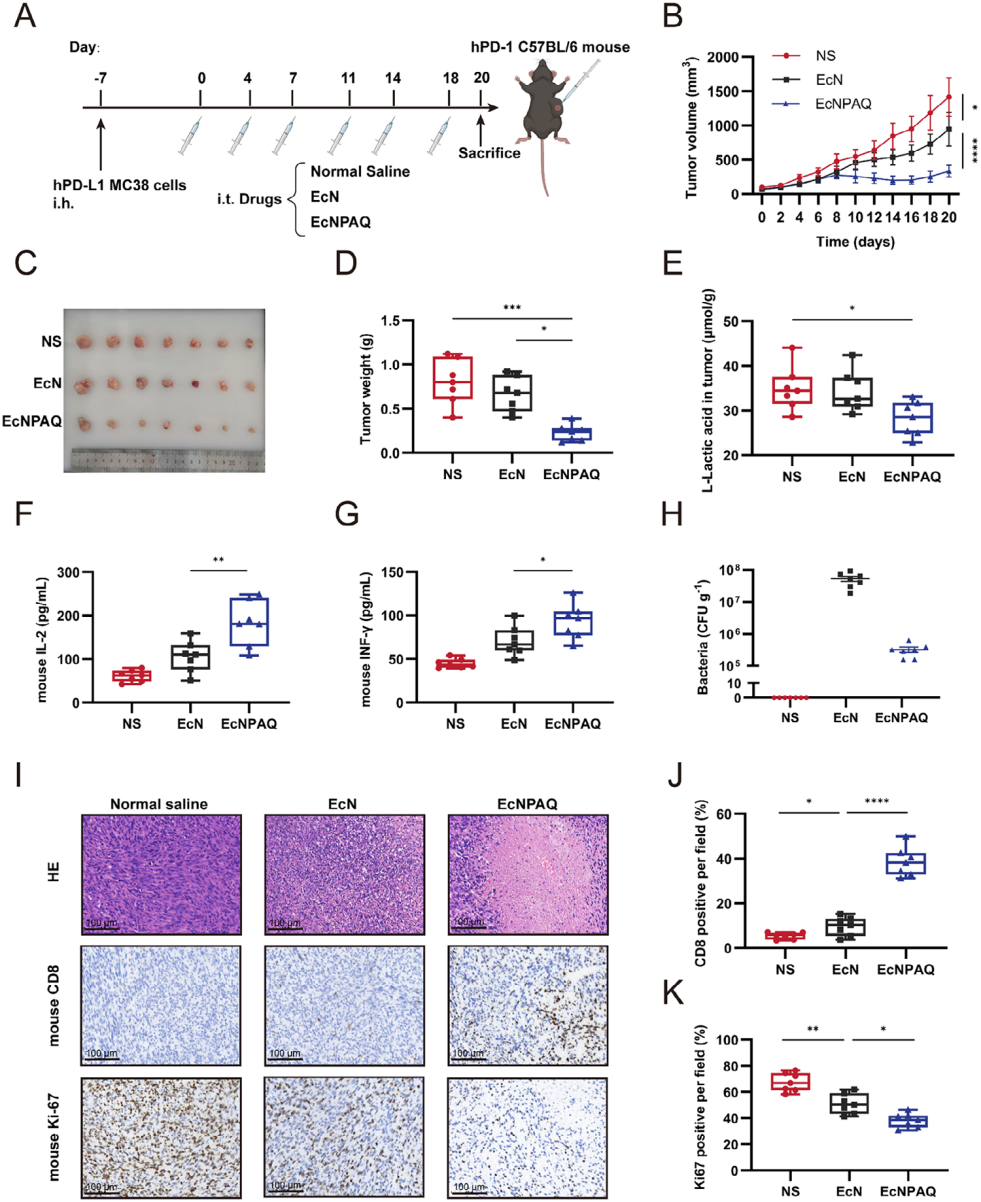
Therapeutic efficacy of EcNPAQ and inhibition of hPD‐L1 MC38 tumor growth in humanized PD‐1 mouse model. A) Schematic illustration of the treatment schedule of the MC38 mouse colon tumor model. C57BL/6 mice were implanted with hPD‐L1 MC38 cells (5 × 10^6^) on the right lower sides of the abdomen on day ‐7. Wen the tumor grew to 100–150 mm^3^, 20 µL Normal saline, EcN or EcNPAQ (5 × 10^8^ CFU mL^−1^ in 20 µL NS) was treated on days 0, 4, 7, 11, 14, and 18 (*n* = 7). Created with BioRender.com. B) The tumor volume of mice treated with different treatment strategies. C) The image of tumors isolated from tumor‐bearing mice on day 20. D) The tumor weight from tumor‐bearing mice on day 20. E) The amount of intratumoral lactate after different treatments. F) The mouse IL‐2 cytokine concentration in serum. G) The mouse INF‐γ cytokine concentration in serum. H) Biodistribution after intratumoral injection of bacteria. Excised tumors were homogenized and plated on LB–agar plates. Colonies were counted to determine CFU/g of tissue. I) H&E and IHC staining of tumor tissues. Scale bar, 100 µm. J) The expression of mouse CD8^+^ cells was confirmed using an immunohistochemistry assay in tumor tissues. K) The expression of mouse Ki67^+^ cells was confirmed using an immunohistochemistry assay in tumor tissues. Data in (A–K) are presented as means ± s.e.m., *n* = 7 mice. *p*‐values were calculated by one‐way ANOVA with Tukey's post‐test. *p < 0.05, **p < 0.01, ***p < 0.001 and ****p < 0.0001.

To further validate the in vivo antitumor activity of EcNPAQ, we conducted therapeutic experiments using a hPD‐1 knock‐in mouse model bearing hPD‐L1 MC38 tumors (**Figure** **5**A). Tumor volume and body weight were monitored bi‐daily (Figure 5B–D; Figure S3, Supporting Information). The results demonstrated that EcN exhibited a certain degree of antitumor activity, while EcNPAQ treatment significantly delayed tumor progression in comparison with the EcN group. It indicated an augmented tumor inhibitory capacity. Body weights remained stable across groups, indicating that there was no systemic toxicity (Figure S4, Supporting Information). In addition, a lower lactate level was detected in the EcNPAQ‐treated group than that in other groups, demonstrating that the EcNPAQ consumed the intratumoral lactate, which can boost the therapeutic efficacy of PD‐L1nb (Figure 5E). Then, 1g of tumor tissue was excised and WB experiments demonstrated that EcNPAQ expressed His‐tag anti‐PD‐L1nb in vivo (Figure S5, Supporting Information).

CD8^+^ T cells have been identified as major effector cells that inhibit the PD‐1/PD‐L1 pathway, thus activating an immune response.^[^ ^53^ ^]^ The infiltration of tumor‐specific CD8^+^ T cells within tumors is a critical indicator of effective immunotherapy, with IFN‐γ serving as an important biomarker for CD8^+^ T cell infiltration.^[^ ^31^, ^54^ ^]^ Additionally, IL‐2, a key growth factor for T cells, is used to assess T cell proliferation and infiltration. We measured serum levels of mouse IL‐2 and mouse IFN‐γ by ELISA to evaluate the treatments' efficacy in blocking the PD‐1/PD‐L1 pathway (Figure 5F,G). Our findings revealed significantly elevated levels of mIL‐2 and mIFN‐γ in the EcNPAQ group compared to the EcN group, suggesting enhanced cytokine secretion related to the antitumor immune response. To rule out systemic dissemination, we evaluated the biodistribution of EcNPAQ. EcN in homogenized tumors, livers and spleens were calculated by plating and qRT‐PCR. It confirmed that bacterial growth was confined to tumors, with no detectable bacteria from the livers and spleens of treated mice (below the limit of detection, ≈1 × 10^3^ colony‐forming units (CFU); Figure 5H; Figure S6, Supporting Information). Because the constant oscillation and lysis of the two strains in EcNPAQ, there are fewer bacteria in the tumor for EcNPAQ than for EcN. Immunohistochemical analysis revealed a substantial augmentation in tumor‐infiltrating mouse CD8^+^ T cells in the EcNPAQ group relative to the EcN group, indicating that enhanced mCD8^+^ T cell infiltration in EcNPAQ‐treated tumor tissues. (Figure 5I,J). Furthermore, mouse Ki67^+^ cells is a critical marker of tumor cell proliferation, present during all active phases of the cell cycle. The expression of mKi67^+^ cells was found to be significantly reduced in the EcNPAQ group in comparison with the EcN group. (Figure 5I,K).

**Figure 6:**
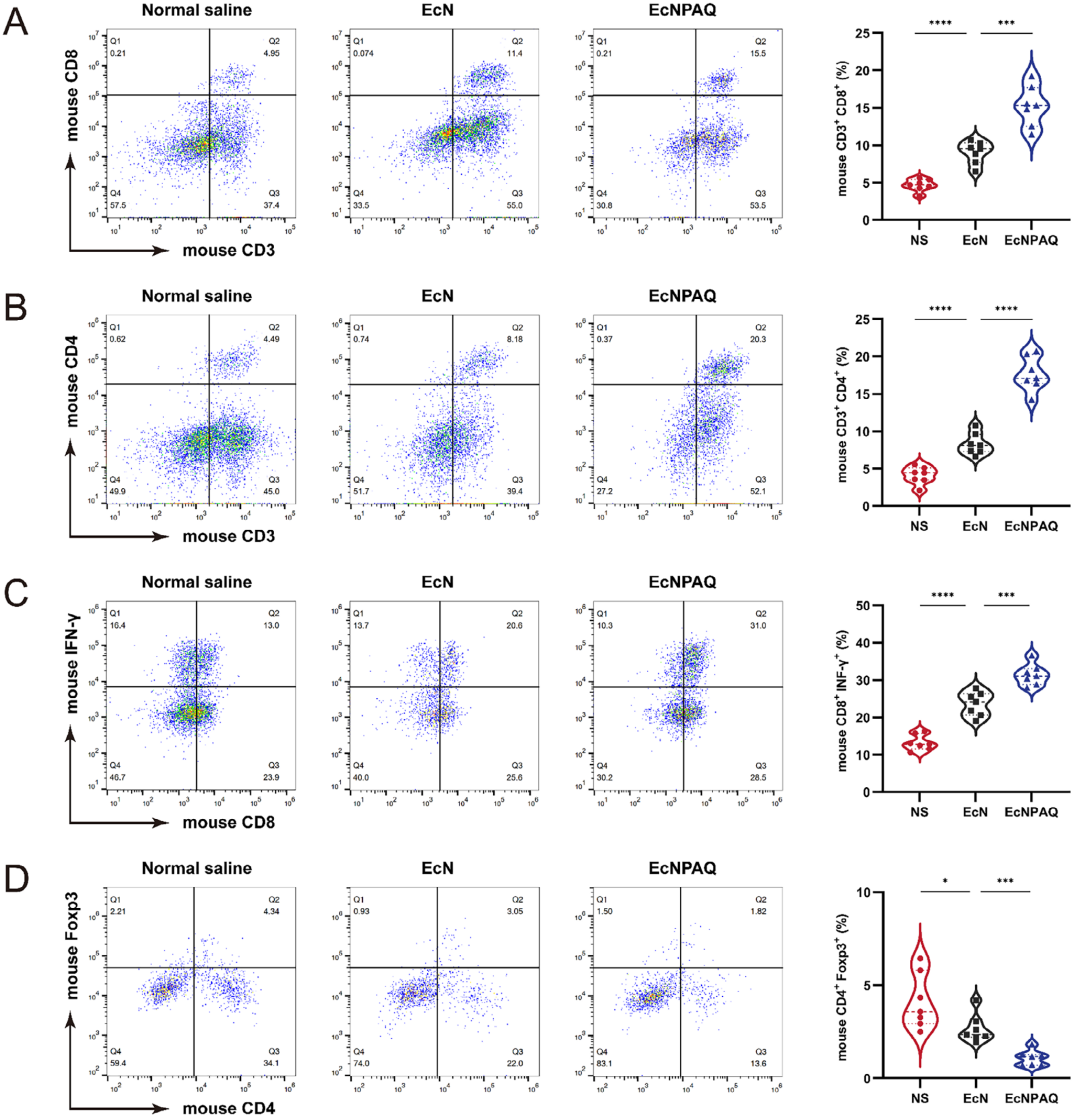
The cell phenotypes in hPD‐L1 MC38 tumor tissues treated with NS, EcN or EcNPAQ. A–D) Tumor‐infiltrating lymphocytes isolated from hPD‐L1 MC38 tumor‐bearing mice on day 20 after the indicated treatments were analyzed by flow cytometry. The representative flow cytometric plots and the frequencies of mouse CD3^+^CD8^+^ T cells A), mouse CD3^+^CD4^+^ T cells B), mouse CD8^+^INF‐γ^+^ T cells C), and mouse CD4^+^Foxp3^+^ T cells D). The detection of intracellular cytokines was achieved through the stimulation of tumor‐infiltrating lymphocytes following *ex vivo* isolation with phorbol 12‐myristate‐13‐acetate (PMA), ionomycin, and protein transport inhibitor (Brefeldin A). Data in (A–D) are presented as means ± s.e.m., *n* = 7 mice. p values were calculated by one‐way ANOVA with Tukey's post‐test. *p < 0.05, **p < 0.01, ***p < 0.001 and ****p < 0.0001.

To further elucidate the in vivo effects observed in the EcNPAQ‐treated group, we digested tumor tissues into single‐cell suspensions and analyzed infiltrating immune cells in the tumors using flow cytometry (Figure S7, Supporting Information). The results indicated that EcN exerted an intrinsic positive effect on the tumor to a certain extent, while EcNPAQ derived a strong systemic antitumor efficacy (**Figure** **6**A–D). The proportion of mCD45^+^ cells increased in tumor tissues from the EcNPAQ group compared to both the NS and EcN groups. Notably, the proportions of mCD3^+^, mCD4^+^, and mCD8^+^ T cells were significantly elevated in the EcNPAQ group. This suggests that EcNPAQ inhibits tumor growth by blocking the PD‐1/PD‐L1 pathway, further promoting T cell infiltration into tumor tissues.^[^ ^55^ ^]^ Additionally, we evaluated the proportion of mIFN‐γ‐producing CD8^+^ T cells in tumor tissue and found a significant increase in this population in the EcNPAQ group compared to the other treatment groups (Figure 6C). Regulatory T cells (T_regs_), marked by Foxp3, facilitate tumor immune evasion by creating a immunosuppressive TME.^[^ ^56^ ^]^ Following incubation with a PE‐labeled anti‐mouse Foxp3 antibody, we observed that the proportion of mFoxp3^+^ T cells was 1.82% in the EcNPAQ group compared to 4.34% in the NS group, indicating that immunosuppressive TME in an activated state (Figure 6D). These findings collectively demonstrate that EcNPAQ augments antitumor immunity. The superior efficacy of EcNPAQ over EcN highlights the therapeutic advantage of its engineered design, likely through synergistic modulation of the tumor immune landscape.

## 6 Antitumor Efficacy of EcNPAQ against HT‐29 Tumors in a Humanized PBMC Mouse Model

**Figure 7:**
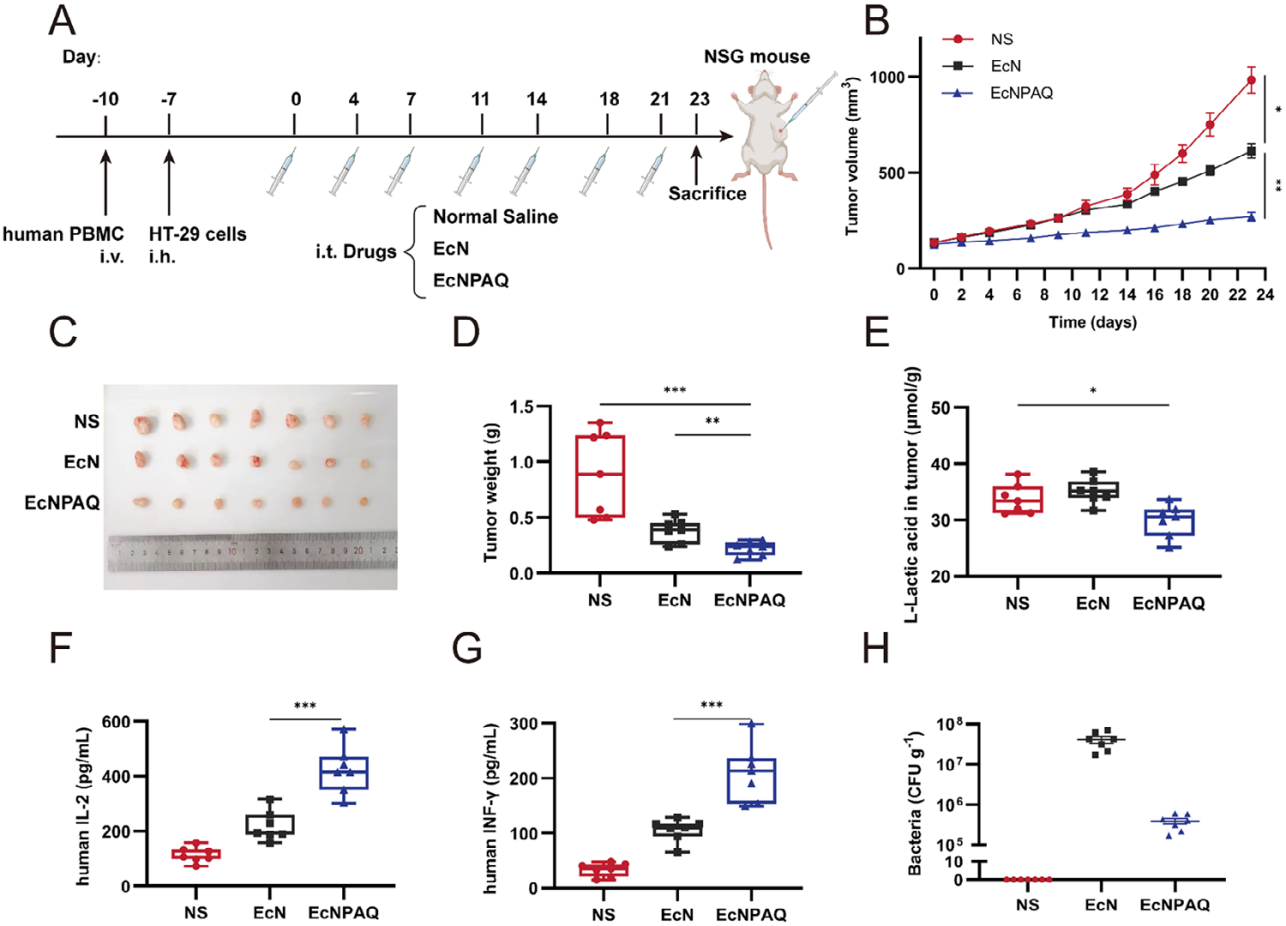
Therapeutic efficacy of EcNPAQ and inhibition of HT‐29 tumor growth in humanized PBMC mouse model. A) Schematic illustration of treatment schedule of HT‐29 mouse colon tumor model. NSG mice were engrafted with human PBMC on day ‐10 and were implanted with human HT‐29 cells (5 × 10^6^) on the right lower sides of the abdomen on day ‐7. Wen the tumor grew to 100–150 mm^3^, 20 µL Normal saline, EcN or EcNPAQ (5 × 10^8^ CFU mL^−1^ in 20 µL NS) was treated on days 0, 4, 7, 11, 14, 18, and 21 (*n *= 7). Created with BioRender.com. B) The tumor volume of mice treated with different treatment strategies. C) The image of tumors isolated from tumor‐bearing mice on day 23. D) The tumor weight from tumor‐bearing mice on day 23. E) The amount of intratumoral lactate after different treatments. F) The human IL‐2 cytokine concentration in serum. G) The human INF‐γ cytokine concentration in serum. H) Biodistribution after intratumoral injection of bacteria. Excised tumors were homogenized and plated on LB–agar plates. Colonies were counted to determine CFU/g of tissue. Data in (A–H) are presented as means ± s.e.m. *n* = 7 mice. *p*‐values were calculated by one‐way ANOVA with Tukey's post‐test. *p < 0.05, **p < 0.01, ***p < 0.001 and ****p < 0.0001.

Although the human PD‐1 mouse model bearing human PD‐L1 MC38 tumor is an effective humanized animal model, syngeneic mouse models have difficulty recapitulating many features of human cancer, such as progressive carcinogenesis and heterogeneity. To overcome these limitations, a classical strategy is to use human tumor cell lines in conjunction with peripheral blood mononuclear cell (PBMC)‐humanized NSG mouse models. To evaluate the therapeutic potential of EcNPAQ, we employed a humanized NSG mouse model engrafted with human PBMC. Three days after PBMC engraftment, the HT‐29 human colorectal tumors were established via subcutaneous injection (**Figure** **7**A). Two weeks later, the proportion of human CD45^+^ cells in the spleen ranges from 52.2% to 63.1% (Figure S8, Supporting Information). This confirmed the successful construction of the humanized mouse model. Mice were intratumorally treated with normal saline (NS), EcN, or EcNPAQ, respectively (Figure 7A). The tumor volume and the body weight were monitored until the mice were euthanized. EcNPAQ treatment significantly delayed tumor progression compared to both EcN and NS groups (Figure 7B–D; Figure S9, Supporting Information). Body weights remained stable across groups, indicating no systemic toxicity (Figure S9, Supporting Information). In addition, we observed that EcNPAQ consumed the intratumoral lactate (Figure 7E), and expressed His‐tag anti‐PD‐L1nb in vivo (Figure S10, Supporting Information).

Cytokines, such as human IL‐2 and human IFN‐γ, function as critical biomarkers of T cell activation. Serum analysis revealed that EcNPAQ‐treated mice had significantly increased levels of hIL‐2 and hIFN‐γ compared to EcN (Figure 7F,G). These findings align with prior studies demonstrating that cytokine‐driven T cell proliferation enhances tumor clearance. To confirm biosafety, homogenized organs and tumors were cultured. EcNPAQ colonization was restricted to tumors, with no detectable dissemination to the liver or spleen (Figure 7H; Figure S11, Supporting Information). Similarly, there are fewer bacteria in the tumor of EcNPAQ group than of EcN group.

**Figure 8:**
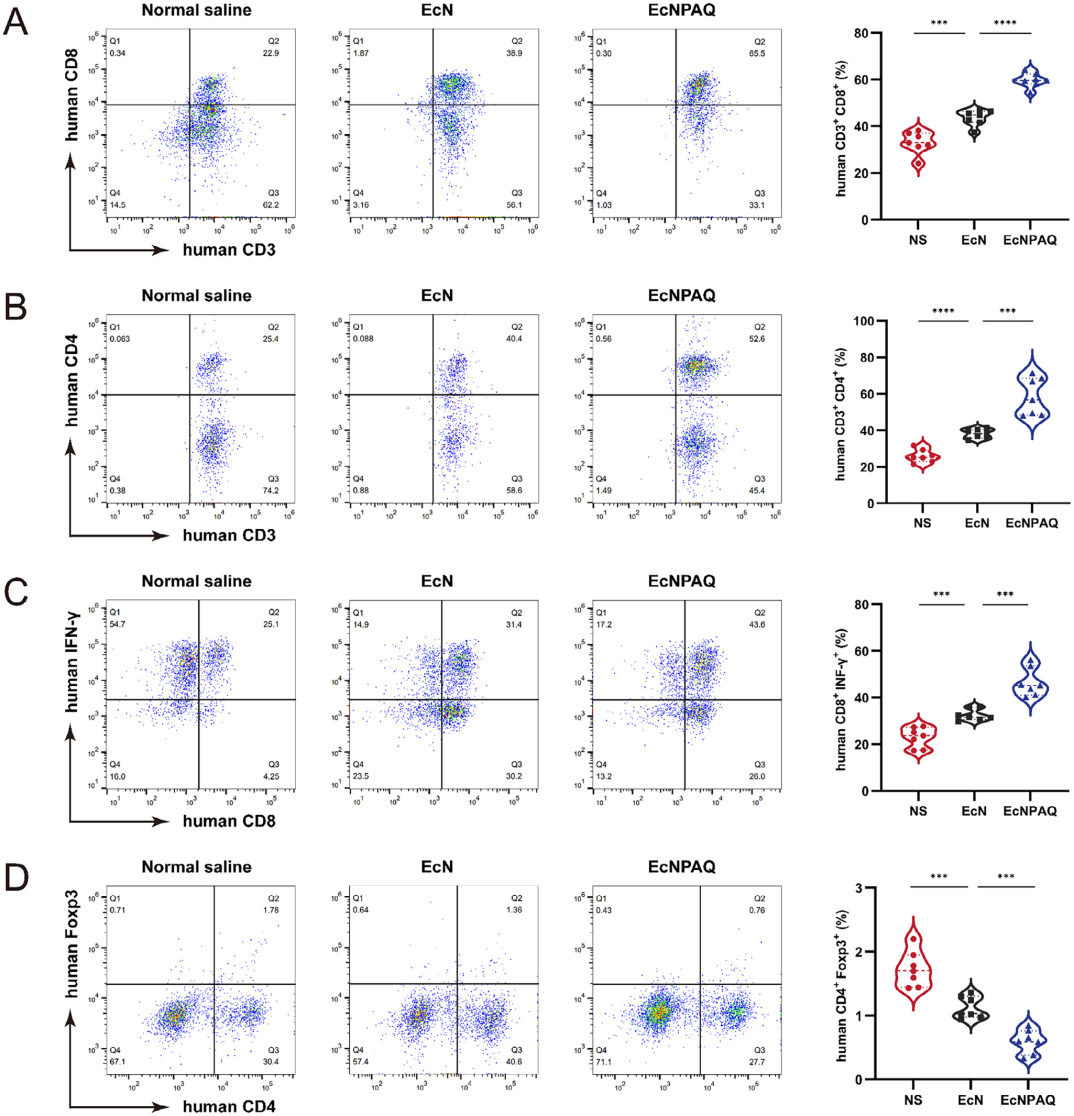
The cell phenotypes in HT‐29 tumor tissues treated with NS, EcN or EcNPAQ. A–D) Tumor‐infiltrating lymphocytes isolated from HT‐29 tumor‐bearing mice on day 23 after the indicated treatments were analyzed by flow cytometry. The representative flow cytometric plots and the frequencies of human CD3^+^CD8^+^ T cells A), human CD3^+^CD4^+^ T cells B), human CD8^+^INF‐γ^+^ T cells C), and human CD4^+^Foxp3^+^ T cells D). The detection of intracellular cytokines was achieved through the stimulation of tumor‐infiltrating lymphocytes following *ex vivo* isolation with phorbol 12‐myristate‐13‐acetate (PMA), ionomycin, and protein transport inhibitor (Brefeldin A). Data in (A–D) are presented as means ± s.e.m., *n* = 7 mice. *p*‐values were calculated by one‐way ANOVA with Tukey's post‐test. *p < 0.05, **p < 0.01, ***p < 0.001 and ****p < 0.0001.

Single‐cell suspensions of dissociated tumors were analyzed by flow cytometry. In humanized PBMC mouse model, the gating strategies of hCD8^+^ T cells, hCD4^+^ T cells, hCD8^+^hINF‐γ^+^ T cells, and hCD4^+^hFoxp3^+^ T cells exhibit consistency with the preceding text, but we used the anti‐human antibodies (Figure S12, Supporting Information). Notably, EcNPAQ expanded hCD8^+^ T cells, hCD4^+^ T cells and hIFN‐γ^+^hCD8^+^ T cells, suggesting that the infiltration of T cells in tumor tissues was significantly enhanced (**Figure** **8**A–C). And it was observed that the proportion of hFoxp3^+^ T cells was 0.76%, while that of NS group was 1.78%, demonstrating that EcNPAQ overcame the immunosuppressive tumor microenvironment (Figure 8D). The improved tumor inhibition observed in the EcNPAQ group is likely due to infiltration and proliferation of intratumoral T cells. Taken together, these results demonstrate that the QS‐based engineered probiotic consortium can generate potent, tumor‐specific adaptive immune responses.

## 7 Conclusion and Discussion

To address challenges such as poor tissue permeability, systemic toxicity, and the immunosuppressive TME in immunotherapy, current research is focused on the engineered single strain for disease diagnosis and treatment.^[^ ^26^, ^57^, ^58^, ^59^ ^]^ We choose to develop an engineered probiotic consortium EcNPAQ. Its advantages include: 1) pH (CadC), hypoxia (FNR), and lactate (LldR) sensors that trigger lysis, thereby combining therapeutic molecules with tumor physiology; 2) the LasR‐plux and TraR‐ptra orthogonal QS systems, which ensure signal specificity and simulate ecological dynamics;^[^ ^10^, ^44^ ^]^ and 3) the release synergistically of two therapeutic molecules, lactate dehydrogenase and anti‐PD‐L1nb. Accordingly, EcNPAQ enables precise delivery of therapeutic molecules in the TME, enhancing both biosafety and specificity. Moreover, optimization of the initial microbial composition using the modified Lotka‐Volterra model improves the stability of oscillations and the feasibility of delivery.

The selection of EcN as a therapeutic chassis was motivated by various biological properties. EcN restructures intestinal microbial ecology to enhance T cell infiltration into tumors and potentiate the efficacy of PD‐1/PD‐L1 checkpoint blockade.^[^ ^29^, ^30^, ^31^ ^]^ Additionally, its anaerobic chemotaxis enables selective colonization of hypoxic tumors, bypassing the reticuloendothelial system (RES) clearance mechanisms that limit drug delivery.^[^ ^60^ ^]^

When constructing engineered microbial consortia, researchers often introduce symbiotic or cooperative interactions to optimize the metabolic flux of the target product and ensure system stability. However, EcNPAQ exemplifies competitive interactions, where the two bacteria mimic predator‐prey dynamics in natural ecosystems, stabilizing lysis to release therapeutic molecules. This approach not only enhances safety in the treatment of human intestinal diseases but also allows for the adjustment of the ratio between the two strains based on specific conditions, enabling better therapeutic outcomes through the synergistic action of the two therapeutic molecules.

High lactate levels in the TME contribute to tumor progression and metastasis, while reducing lactate concentrations improves cytotoxic T cell function, thereby enhancing the sensitivity of PD‐L1 immune therapies.^[^ ^61^, ^62^ ^]^ To further enhance immunotherapy, we designed EcNPAQ to precisely produce ldhA and anti‐PD‐L1nb, synergistically targeting immune evasion and metabolic immune suppression. Based on these findings, we hypothesize that EcNPAQ could promote T cell infiltration into tumor tissues and inhibit tumor growth.

To test this hypothesis, we evaluated the therapeutic effect of EcNPAQ in the humanized PD‐1 mouse model and humanized PBMC mouse model. There is a critical need to evaluate the therapeutic efficacy of an engineered bacterial delivery system in humanized mouse models. Although these models have a limited immune repertoire and cannot fully replicate the complexity of the human immune system, they also provide a more effective platform for evaluating therapeutic efficacy.^[^ ^63^ ^]^ Tumor‐infiltrating lymphocytes (TILs) are crucial for mediating anti‐tumor immune responses, with their density and distribution impacting patient prognosis across various cancers. The increased ratio of CD8^+^ T cells and CD4^+^ T cells in the EcNPAQ group initiated a strong T cell‐mediated anti‐tumor response.^[^ ^64^ ^]^ Additionally, elevated CD8^+^IFNγ^+^ T cell levels correlated with improved prognosis in CRC patients. A significant increase in IFNγ^+^ T cells also facilitated the polarization of macrophages from the M2 to the M1 phenotype, further enhancing dendritic cell maturation and anti‐tumor immunity. Furthermore, a reduction in CD4^+^Foxp3^+^ regulatory T cells (Tregs) in the tumor tissues indicated a suppression of the tumor's immunosuppressive microenvironment, enhancing the overall anti‐tumor immune response.^[^ ^56^ ^]^ These results mirror findings in PD‐1/PD‐L1 blockade studies, where T_regs_ depletion synergizes with CD8^+^ T cell infiltration to overcome immune evasion.

Although our experiments have demonstrated the anti‐tumor efficacy of EcNPAQ, its therapeutic effects have yet to be validated in situ tumor models by cancer cells with high PD‐1 expression and oral administration. Moreover, we will evaluate the therapeutic efficacy of EcN expressing only anti‐PD‐L1nb, EcN expressing only LdhA, and EcNPAQ. It can demonstrate that in engineered bacterial therapy, lactate degradation combined with PD‐L1nb also has the synergistic effects reported in current articles.^[^ ^16^, ^65^, ^66^ ^]^ Additionally, we will optimize the balance between the release of lactate dehydrogenase and anti‐PD‐L1nb to further enhance the combined therapeutic effect.

This highly modular system serves as a durable and stable platform. In the future, this platform could be adapted to respond to disease signals beyond oncology, providing precise therapeutic solutions through integrated genetic circuits. However, this presents challenges in terms of targeted bacterial delivery and minimizing in vivo toxicity.^[^ ^67^ ^]^ For example, EcNPAQ could be engineered to respond to thiosulfate signals, enabling the production of immunomodulatory proteins, enzymes, or anti‐inflammatory cytokines for the treatment of intestinal inflammation.^[^ ^68^ ^]^ Another promising application of this consortium system is the delivery of ICIs and tumor neoantigens, which could enable the development of personalized cancer vaccines using engineered bacteria as vectors.^[^ ^57^, ^69^ ^]^ By programming microbial vectors and combining them with other immunotherapies, EcNPAQ could provide a reliable and efficient platform to eradicating primary and metastatic solid tumors. Probiotic therapy shows strong potential for rapid clinical translation, facilitated by approaches such as enteric‐coated capsules, magnetic or sonogenetics‐controlled targeted delivery.

In conclusion, we anticipate that the engineered probiotic consortium will open new avenues for precision medicine, ushering in the era of controlled and genetically engineered probiotics therapies.

## 8 Experimental Section

### Strains and Medium

Full strain information was provided in Table S1 (Supporting Information). DH5α and BL21(DE3) was purchased from Transgen Biotech, China. *E. coli* Nissle 1917 (EcN) was stored in our laboratory. All bacteria were cultured in LB media (Oxoid) with appropriate antibiotic selection (100 µg mL^−1^ ampicillin, 50 µg mL^−1^ kanamycin) at 37 °C.

### Gene Deletions

The gene *ldhA* was deleted using the λ‐Red recombination system. Linear DNA were PCR amplified and electroporated into EcN carrying pKD46 plasmid. Bacteria were recovered and plated. Chromosomal deletions were verified by PCR and sequencing.

### Plasmid Constructions

The hypoxia, pH, and lactate biosensors were constructed by synthesizing promoters from Genewiz and were cloned in front of the GFP gene of the pET‐22b plasmid. The QS system was derived from the plasmids pET‐22b and pRSFDuet‐1. The LuxI, LasI, TraI, RpaI, and RhlI synthases were expressed with a constitutive promoter J23119 in the pRSFDuet‐1 plasmid. The pLux, pLas, pTra, pRpa, and pRhl sensors were assembled upstream of GFP, respectively. In order to provide orthogonal verification, the LuxR, LasR, TraR, RpaR, and RhlR receptors were expressed constitutively with the promoter J23119 and constructed with five different sensors in the pRSFDuet‐1 plasmid. In the context of the microbial consortium, the pRSFLA plasmid was constructed by cloning the L‐lactate acid biosensor and degradation enzyme, as well as the pJ23114 promoter, upstream of TraR, LuxI, and GFP into a pPSFDuet‐1 plasmid. The pETLA lysis plasmid was constructed by cloning a pH biosensor inducing the ccdB toxin protein and the pRpa promoter inducing the ccdA antitoxin protein into a pET22b plasmid. The pRSFPD therapeutic plasmid was constructed by cloning the pLux promoter upstream of ccdB and a hypoxia biosensor upstream of the anti‐PD‐L1nb with a His‐tag. The pETPD plasmid was constructed by cloning the pJ23114 promoter upstream of TraI, LuxR and RFP into a pET22b plasmid. All plasmids were constructed using a MultiS One Step Cloning kit (C113–01, Vazyme) and transformed into *E. coli* DH5α and then into EcN competent cells. All relevant genetic components have been confirmed by sequencing.

### Characterization of Biosensors In Vitro

Each bacterial strain was grown in liquid culture overnight, and then used to inoculate induction experiments. For hypoxia biosensors, each strain was grown overnight in normoxic condition and was cultured in hypoxic fermenters at 37 °C. For pH biosensors, each variant was cultured in 1 L fermenters with a pH ranging from 5.0–7.0. For lactate biosensors, strain was cultured overnight in 50 mL LB broth with the relevant antibiotics and at lactic acid concentrations of 0.01, 0.1, 1, 2, 5, 8, and 10 mM. Negative controls of EcN cells transformed pET‐22b plasmid were grown in the same conditions. Following a period of 16–20 h of growth, fluorescence and absorbance data were collected using the MicroPlate Reader.

### Characterization of QS System In Vitro

^−^ The overnight‐cultured strains were inoculated into 200 mL LB broth and incubated at 220 rpm min^−1^ and 37 °C. Upon reaching an OD_600_ of 0.1, the cultures were transferred to 96‐well plates. EcN cells with QS receptors and sensors were induced by two different signals in different concentrations ranging from 10^−4^ to 10^−13^ M. Following a 3 h incubation period, fluorescence and absorbance data were collected in 96‐well plates using the MicroPlate Reader.

### Fluorescence Data Analysis

All biosensor in vitro fluorescence signals were calculated by dividing the raw GFP pixel intensity by the OD_600_ value, both obtained from plate reader data. The background fluorescence signal (plasmid pET‐22b, serving as a control for the same strain) was subtracted. The fluorescence signals were quantified as the ratio between the induced and non‐induced conditions. The triplicate values were averaged.

### Characterization of Lactate Consumption

EcN which expresses lactate dehydrogenase (LdhA) in the plasmid pET‐22b, was cultured in anaerobic 50 mL LB broth. After 2 h, the concentration of lactic acid was measured by using a lactate assay kit (Nanjing Jiancheng Bioengineering Institute, China). The UV−vis absorbance spectra were analyzed by using UV−vis spectroscopy (Shanghai INESA, China).

### Characterization of ccdA and ccdB

The plasmid pET‐22b, which constitutively expresses ccdB and expresses ccdA with the arabinose‐induced araBAD promoter, was constructed and transformed into EcN. The bacterium was cultured overnight and 100 µL of the dilution was applied to plates containing 0%, 0.5%, 1%, 1.5%, 2%, and 2.5% arabinose. The number of colonies was counted after 12 h.

### Mathematical Model

To describe the dynamic behavior of the predator‐prey system, we developed a modified Lotka‐Volterra equation. Let x_1_ represent the number of EcNPD cells, and *x*_2_ represent the number of EcNLA cells. The growth rate of EcNPD cells in an anaerobic environment with pH 5.5 and a 2 mM lactate concentration is denoted by r_1_. Similarly, the degradation rate of EcNLA cells under the same conditions is denoted by r_2_. R represents the competitive death that occurs when both EcNLA and EcNPD populations reach the maximum environmental capacity. λ_1_ defines the ability of EcNPD cells expressing the ccdB toxin protein to promote mortality in the presence of sufficient LuxI. Conversely, λ_2_ defines the ability of EcNLA cells expressing the ccdA antitoxin protein to enhance survival against the ccdB toxin protein when sufficient TraI is present.

(1) $$\frac{dx_{1}}{dt}=r_{1}x_{1}\left(1-\frac{x_{1}}{R}\right)-\lambda_{1}x_{1}x_{2}$$

(2) $$\frac{dx_{2}}{dt}=x_{2}\left(-r_{2}+\lambda_{2}x_{1}\right)$$

### Model Parameter Values

*r*_1_ (The rate of EcNPD cell independent growth) 0.6; *r*_2_ (The rate of EcNLA cell independent lysis) 0.2; R (Competitive death rate) 30 000; *λ*_1_ (The rate of EcNPD cell lysis with EcNLA) 0.0004; *λ*_2_ (The rate of EcNLA cell survival with EcNPD) 0.0004.

### Characterization of the Microbial Consortium In Vitro

The EcNLA and EcNPD strains were cultured in 1 L fermenters and sampled at hourly intervals over a 72 h period. Additionally, samples were plated on LB agar plates with 100 µg mL^−1^ ampicillin and 50 µg mL^−1^ kanamycin, allowing for the calculation of c.f.u values for green and red fluorescence the following day. And it was sampled at different times (0, 6, 12, 24, 36, 48, 60, and 72 h), with one portion measuring anti‐PD‐L1 nb production by ELISA and the other portion of the sample used to take images by Laser Confocal Scanning Microscope (Leica Stellaris 8). The fluorescent areas in the images were quantified by ImageJ.

### Analysis the Secretion of Anti‐PD‐L1nb by ELISA

The anti‐PD‐L1nb gene containing the 6 × His tag was inserted into the protein expression backbone pRSFDuet‐1 and then transformed into the EcN strain. The standard curve was obtained using purified anti‐PD‐L1nb protein. The anti‐PD‐L1nb was diluted to 100, 50, 25, 12.5, and 0 ng mL^−1^. Subsequently, 100 µL of diluted anti‐PD‐L1nb was added to the ELISA plate, and the plate was then incubated overnight at 4 °C and washed three times with 250 µL of TBST buffer. After blocking with 200 µL of 5% bovine serum albumin (BSA) in TBST for 1 h at 37 °C and subsequent washing steps, goat anti‐mouse IgG conjugated to horseradish peroxidase (HRP) (diluted 1:2000 in TBST with 5% BSA) was added to each well, and the plate was incubated for 1 h at 37 °C and subsequently washed. After the washing steps, 200 µL of TMB was added to each well, and the plate was incubated for 15 min at 37 °C. Sulfuric acid (2 M) was added to each well to stop the reaction, and the OD_450_ was then read using a microplate reader. For the assessment of anti‐PD‐L1nb secretion by engineered EcN, the samples were centrifuged at 5000 rpm for 10 min to collect the culture supernatant. The medium was filtered through 0.45‐mm syringe filters, and 100 µL of the prepared medium samples were added to the ELISA plate. The plate was then incubated overnight at 4 °C, and the level of anti‐PD‐L1nb in LB medium was detected by ELISA.

### Mammalian Cell Culture

The THP‐1, HT‐29, and hPD‐L1 MC38 cell lines were stored in our laboratory. Mammalian cells were cultured in Roswell Park Memorial Institute (RPMI) 1640 medium (Gibco) with 10% fetal bovine serum (FBS, Gibco) and 1% penicillin–streptomycin (Solarbio) and placed inside a tissue culture incubator at 37 °C maintained at 5% CO_2_. Trypsin was purchased by Invitrogen (USA). Moreover, to evaluate the generation and exhaustion of lactate in glucose‐containing medium, HT‐29 cells were seeded on the down chamber of a 12‐well transwell (5 × 10^4^ cells per well) and cultured by RPMI 1640 medium (0.5 mL per well) containing 10% FBS for 24 h. Then, the bacterial supernatants with concentration of 5 × 10^7^ CFU mL^−1^ were added to the top chamber of the transwell. After 24 h of incubation, the concentration of lactate was detected by using a lactate assay kit.

### In Vitro Phagocytosis

EcNLA and EcNPD strain were grown to mid‐log phase in a shaking incubator at 37 °C and co‐cultured for 12, 24, 36, and 48 h, respectively. The EcNLA culture was added 10^−6^ M TraI signal, while the EcNPD culture was added 10^−6^ M LuxI signal. After 48 h, the supernatants from both the EcNLA and EcNPD groups were collected. Cultures were then centrifuged at 10 000 r.p.m. for 5 min and supernatants were filtered through a 0.45‐µm filter and stored at −80 °C. THP‐1 cells were detached and seeded onto a transparent 12‐well tissue culture plate at 1 × 10^5^ cells per well. M0‐ polarized THP‐1 cells were generated by treatment with 50 ng mL^−1^ phorbol myristate acetate (PMA, Sigma–Aldrich) for 24 h. HT‐29 tumor cells were incubated for 10 min with 4 µM DiD Perchlorate solution (Yeasen, China). Labeled HT‐29 cells were pretreated 1:1 with supernatants of bacterial lysates or with anti‐PD‐L1 monoclonal antibody or sterile PBS. After 3.5 h co‐incubation, residual HT‐29 cells were washed and THP‐1 cells were stained with Hoechst 33 342 (Solarbio, China). Confocal microscopy photos were observed by Laser Confocal Scanning Microscope (Leica Stellaris 8). The ratio of DiD^+^ THP‐1 cells to total Hoechst 33 342^+^ THP‐1 cells was counted for each field to score phagocytosis.

### Animal Tumor Models

The human PD‐1 knock‐in female mice and NOD.Cg‐*Prkdc^scid^Il2rg^em1Smoc^* female mice were purchased from Shanghai Model Organisms Center, and maintained under specific pathogen‐free conditions. All animal experiments were approved by the Institutional Animal Care and Use Committee (Tianjin University, protocol TJUE‐2024‐354 and protocol TJUE‐2025‐176). The protocols require animals to be euthanized when tumor burden reaches 2 cm in diameter or under veterinary staff recommendation. Mice were randomized into various groups in a blinded manner. In humanized PD‐1 mouse model, hPD‐L1 MC38 mouse colorectal cells (2 × 10^6^ cells/mouse) were subcutaneously injected into the right flank of human PD‐1 knock‐in mice. In humanized PBMC mouse model, 6–8‐week‐old NSG mice were intravenously injected with 10^6^ human PBMC. HT29 human colorectal cells (2 × 10^6^ cells/mouse) were subcutaneously injected into the right flank of NSG mice. Tumor cells were injected at a volume of 100 µL per mouse. The mice were divided randomly into three groups, and were injected 20 µL normal saline, EcN or EcNPAQ every 3‐4 days. Tumors were grown to an average of ≈100–150 mm^3^ before bacterial injections. Tumor volume was quantified using calipers to measure the length, width of each tumor (V = L×W×W×0.5). Values were computed as the mean±s.e.m.

### Bacterial Administration for In Vivo Experiments

Bacterial strains were grown overnight in LB medium, which contained the appropriate antibiotics. A 1:100 dilution into medium with antibiotics was started the day of injection and grown to an OD_600_ of ≈0.1. Bacteria were spun down and washed three times with sterile normal saline before injection into mice. EcNLA and EcNPD were mixed in a 1:100 ratio. Intratumoral injections of bacteria were performed at a total concentration of 5 × 10^8^ CFU mL^−1^ in NS at a volume of 20 µL per tumor injected every 3‐4 days. Mice were euthanized on day 20 or 23.

### Biodistribution and In Vivo Bacterial Dynamics

Tumors, livers and spleens were weighed, clipped and homogenized. They were serially diluted, plated on LB–agar plates and cultured at 37 °C overnight. Colonies were counted (limit‐of‐detection 10^3^ CFU g^−1^) and counted as CFU/g of tissue. For qRT‐PCR, primers were designed for the *fimA* gene of type I pili, which was specific to *Escherichia coli*. The forward primer sequence is 5′‐ATACTACGACGGTAAATGGT‐, and the reverse primer sequence is 5′‐TACATCAGTATGCGTAGCAT‐. According to NCBI BLAST, they were specific to *E. coli*. Then EcN absolute quantification was counted by log_10_C per gram tissues. C was the copy number.

### Western Blot Analysis

1 g tumor tissues underwent homogenization and lysis on ice with cell lysis buffer for western (Solarbio, Beijing, China) for 0.5 h. The proteins that had been dissolved in the supernatants were gathered, analyzed using SDS‐PAGE, and then transferred onto polyvinylidene difluoride membranes. Detection was conducted using the ECL chemilumi‐nescence detection kit by Solarbio. HRP‐conjugated His‐Tag Monoclonal antibody (cat#HRP‐66005, Proteintech Group, Inc.) were used.

### IL‐2 and INF‐γ Secretion Assays

In humanized PD‐1 mouse model, enzyme‐linked immunosorbent assay kits (Mouse IL‐2 ELISA kit, cat#E‐EL‐M0042, Elabscience) (Mouse IFN‐γ ELISA kit, cat#E‐EL‐M0048, Elabscience) were used to detect the secretion of mIL‐2 and mINF‐γ according to the manufacturer's instructions. In humanized PBMC mouse model, ELISA kits (Human IL‐2 ELISA kit, cat#E‐EL‐H0099, Elabscience) (Human IFN‐γ ELISA kit, cat#E‐EL‐H0108, Elabscience) were used to detect the secretion of hIL‐2 and hINF‐γ. Briefly, supernatant of serum with different treatment was collected and centrifuged (500 g) for 15 min. Then, the supernatant was subjected to ELISA assay.

### Hematoxylin and Eosin (H&E) Staining and Immunohistochemistry (IHC) Staining

Tumors were surgically removed, washed with phosphate‐buffered saline (PBS), fixed in 4% formaldehyde, embedded in paraffin, and then sectioned into slices 5 µm thick. One set of paraffin sections was stained with hematoxylin and eosin (H&E) staining. Another Immunohistochemistry (IHC) staining images of CD8^+^ cells (anti‐CD8α antibody, cat#17 147 from Abcam) and Ki67^+^ cells (anti‐Ki67 antibody, cat#279 653 from Abcam) in tumor tissues.

### Cell phenotype Analysis in Tumor Tissues by Flow Cytometry

Tumor tissues were cut into pieces and digested by 1 mL of collagenase(1 mg mL^−1^), hyaluronidase(0.1 mg mL^−1^) and DNase I (0.1 mg mL^−1^) on a shaker at 80 rpm and 37 °C for 1 h. The obtained cell suspension was ground and filtered with 70 µm cell strainers to clean tubes. In humanized PD‐1 mouse model, according to the instructions of manufacturer, cells were labeled with PE/Cy7 anti‐mouse CD45 antibody (cat#103 113), FITC anti‐mouse CD3 antibody (cat#100 203), PE anti‐mouse CD8α antibody (cat#100 707), APC anti‐mouse CD4 antibody (cat#100 411), APC anti‐mouse IFN‐γ antibody (cat#505 809) and PE anti‐mouse FOXP3 antibody (cat#126 403), respectively. In humanized PBMC mouse model, the same anti‐mouse antibodies were replaced with anti‐human antibodies (PE/Cy7 anti‐human CD45 antibody, cat#304 015, FITC anti‐human CD3 antibody, cat#300 405, PE anti‐human CD8α antibody, cat#301 007, APC anti‐human CD4 antibody, cat#300 514, APC anti‐human IFN‐γ antibody, cat#502 511, PE anti‐human FOXP3 antibody, cat#320 107). And PE/Cy7 anti‐human CD45 antibody (cat#304 015) and PerCP anti‐mouse CD45 antibody (cat#103 130) were used to validate the successful construction of PBMC humanized mouse model. All antibodies were purchased from BioLegend. After filtered with 70 µm strainers to clean tubes, cell phenotypes were analyzed by flow cytometry (CytoFLEX, Beckman, USA). The results were analyzed by FlowJo 10.9.0.

### Statistical Analysis

Statistical tests were performed using either GraphPad Prism 10.0 (Student's t‐test and analysis of variance (ANOVA)) or Microsoft Excel. The details of the statistical tests are indicated in the respective figure legends. When data were approximately normally distributed, values were compared using a Student's t‐test, one‐way ANOVA for a single variable or a two‐way ANOVA for two variables with Bonferroni correction for multiple comparisons. Statistical significance was determined at a threshold of *p* < 0.05. The following notation was used to denote significance levels: (n.s.) for *p* > 0.05, * for *p* < 0.05, ** for *p* < 0.01, *** for *p* < 0.001, and **** for *p* < 0.0001. Results with p values above 0.05 were considered not significant (n.s.).

## Conflict of Interest

The authors declare no conflict of interest.

## Supporting information



Supporting Information

## Data Availability

The data that support the findings of this study are available from the corresponding author upon reasonable request.
