# Supplementary material for: An Engineered Probiotic Consortium Based on Quorum‐Sensing for Colorectal Cancer Immunotherapy
Source: Adv Sci (Weinh). 2025 Sep 26;12(46):e12744. doi: 10.1002/advs.202512744 (PMC12697889; doi:10.1002/advs.202512744)
Supplement: Supplementary file 1 — Supporting Information [file ADVS-12-e12744-s001.docx]

**Supporting Information**

**An Engineered Probiotic Consortium Based on Quorum-sensing for Colorectal Cancer Immunotherapy**

*Yufei Guo, Mengxue Gao, Lina Wang, Haibin Yuan, Jianan Yin, Jiahao Wu, Xinran Gao, Zhixin Zhu, Yan Zhang, Zichen Wang, He Huang*, Guangbo Kang**

Y. G. Author 1, M. G. Author 2, L. W. Author 3, H. Y. Author 4, J. Y. Author 5, J. W. Author 6, X. G. Author 7, Z. Z. Author 8, Y. Z. Author 9, Z. W. Author 10, H. H. Author 11, G. K. Author 12

School of Synthetic Biology and Biomanufacturing, State Key Laboratory of Synthetic Biology, Tianjin Key Laboratory of Biological and Pharmaceutical Engineering, Tianjin University, Tianjin, China

E-mail: [huang@tju.edu.cn](mailto:huang@tju.edu.cn); [guangbo_kang@tju.edu.cn](mailto:guangbo_kang@tju.edu.cn).

Funding: The present study was supported by grants from the National Key Research and Development Project (Grant No. 2024YFA0918500)

Keywords: microbial consortium, engineered probiotic, quorum sensing, immunotherapy, colorectal cancer

**Table of Contents**

Figure S1………………………………………………………..…………………..3

Figure S2……………………………………………………………..……………..5

Figure S3……………………………………………………………..……………..6

Figure S4……………………………………………………………..……………..7

Figure S5………………………………………………………………..…………..8

Figure S6………………………………………………………………..…………..9

Figure S7…………………………………………………………………………..10

Figure S8…………………………………………………………….……………..11

Figure S9…………………………………………………………………………..12

Figure S10…………………………………………………………………………13

Figure S11…………………………………………………………………………14

Figure S12…………………………………………………………………………15

Table S1……………………....……………………………………………………16

Figure S1


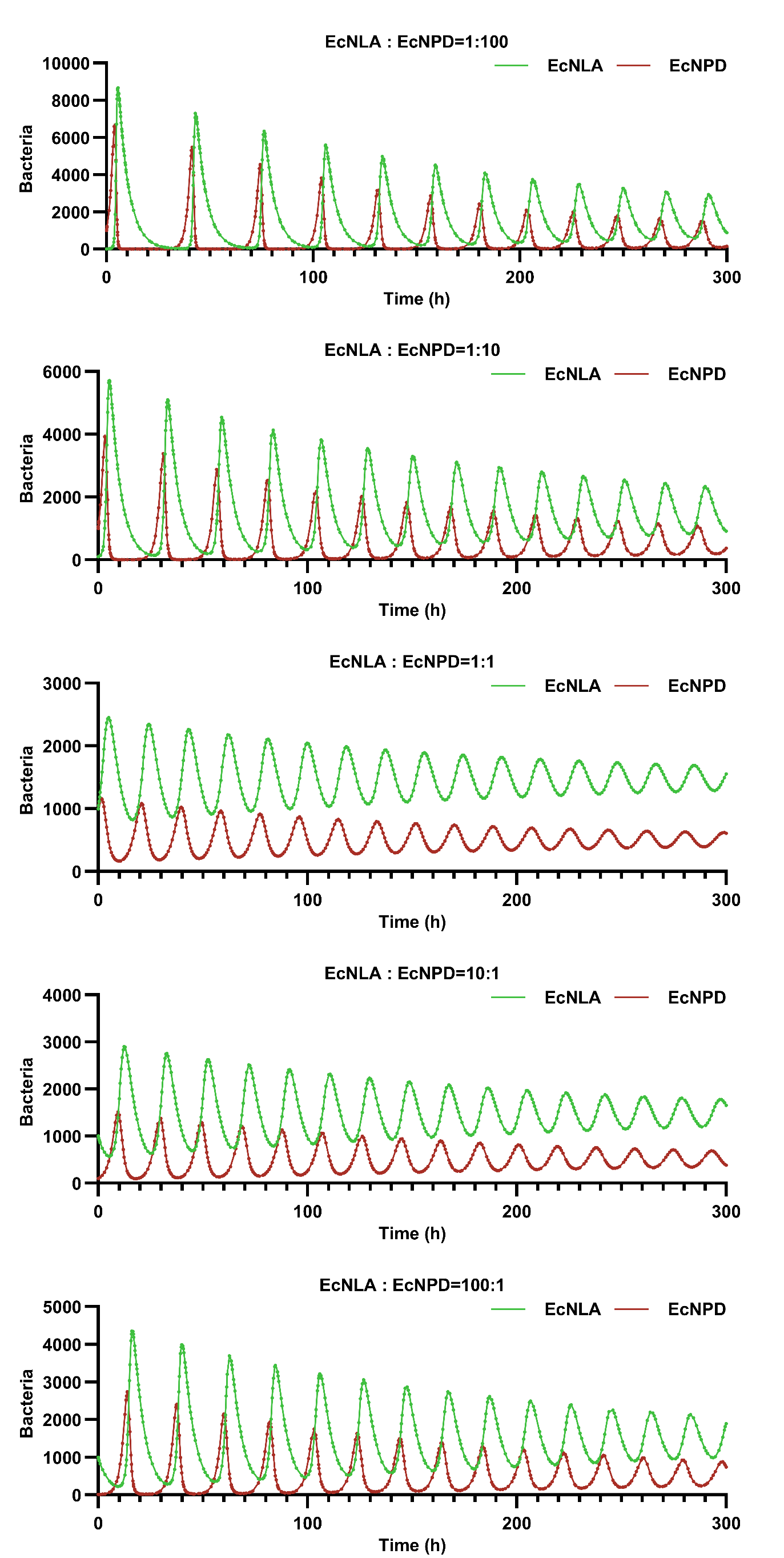


**Figure S1.** The estimated cell population trajectory reveals that the stable oscillation of the predator-prey system is influenced by different initial conditions. Specifically, when the initial concentration ratio of EcNLA to EcNPD is 1:100, the system exhibits greater stability and efficiency.

Figure S2


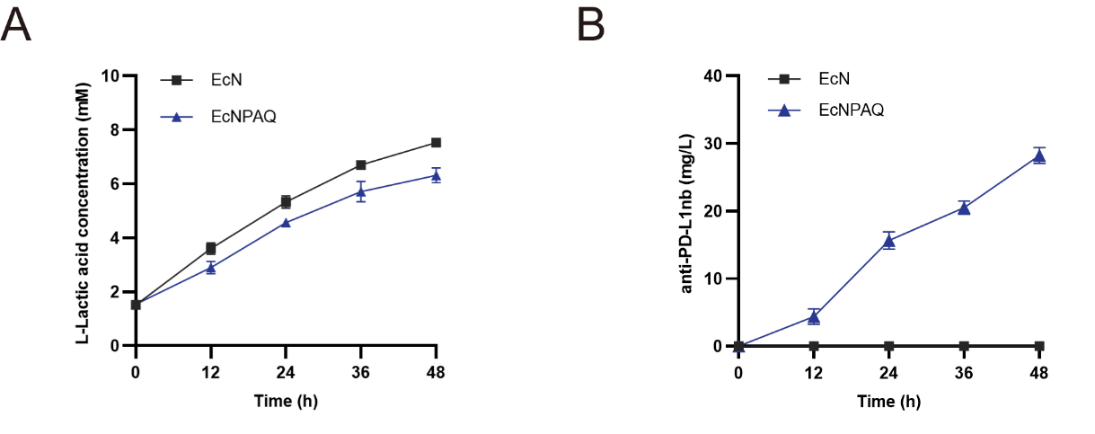


**Figure S2.** HT-29 cells were co-cultured with EcN or EcNPAQ at an initial ratio of 1:100 in a Transwell chamber for 48 hours. Every 12 hours, 100 μL of bacterial culture was sampled to measure lactate concentration or PD-L1 nanobody (PD-L1nb) concentration. Although lactate levels increased over time, the EcNPAQ group effectively reduced lactate concentration (A). Additionally, PD-L1nb production in the EcNPAQ group increased progressively over time (B).

Figure S3


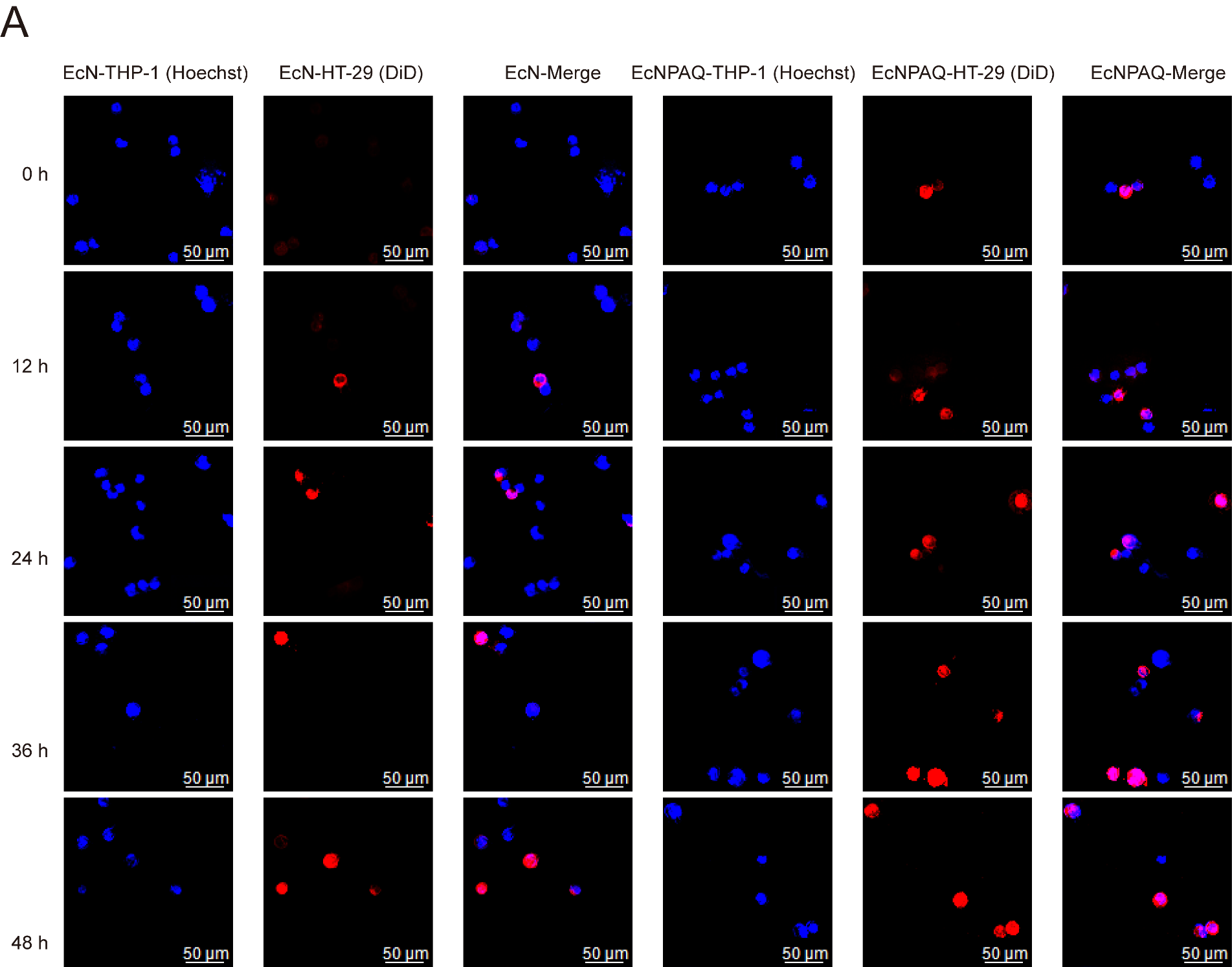


**Figure S3.** Microscopic visualization of the phagocytosis of NS, EcN and EcNPAQ pretreated at different co-culture time.

Figure S4


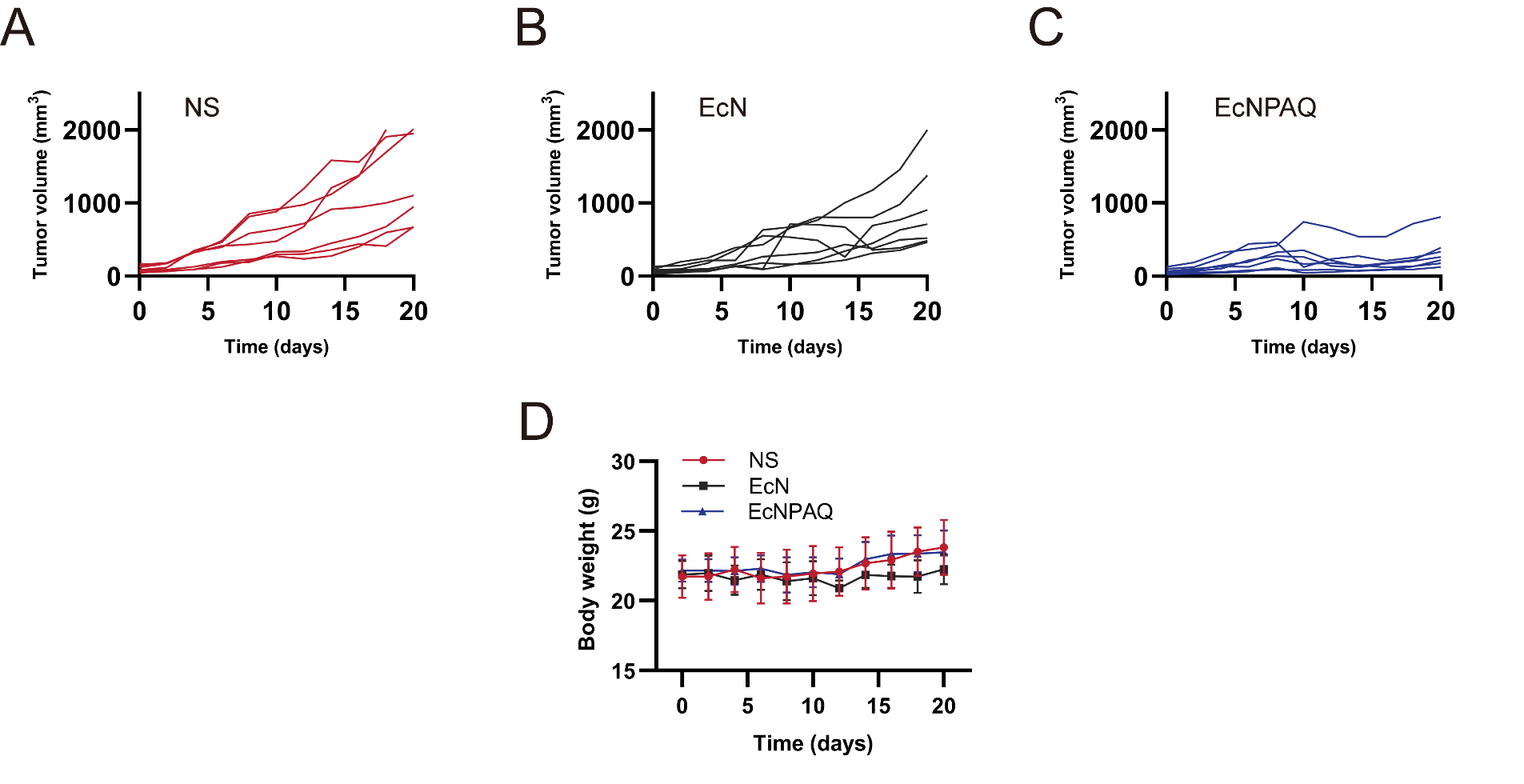


**Figure S4.** In humanized PD-1 mouse model, the tumor growth curves of individual mice and the body weight of three groups. n=7.

Figure S5


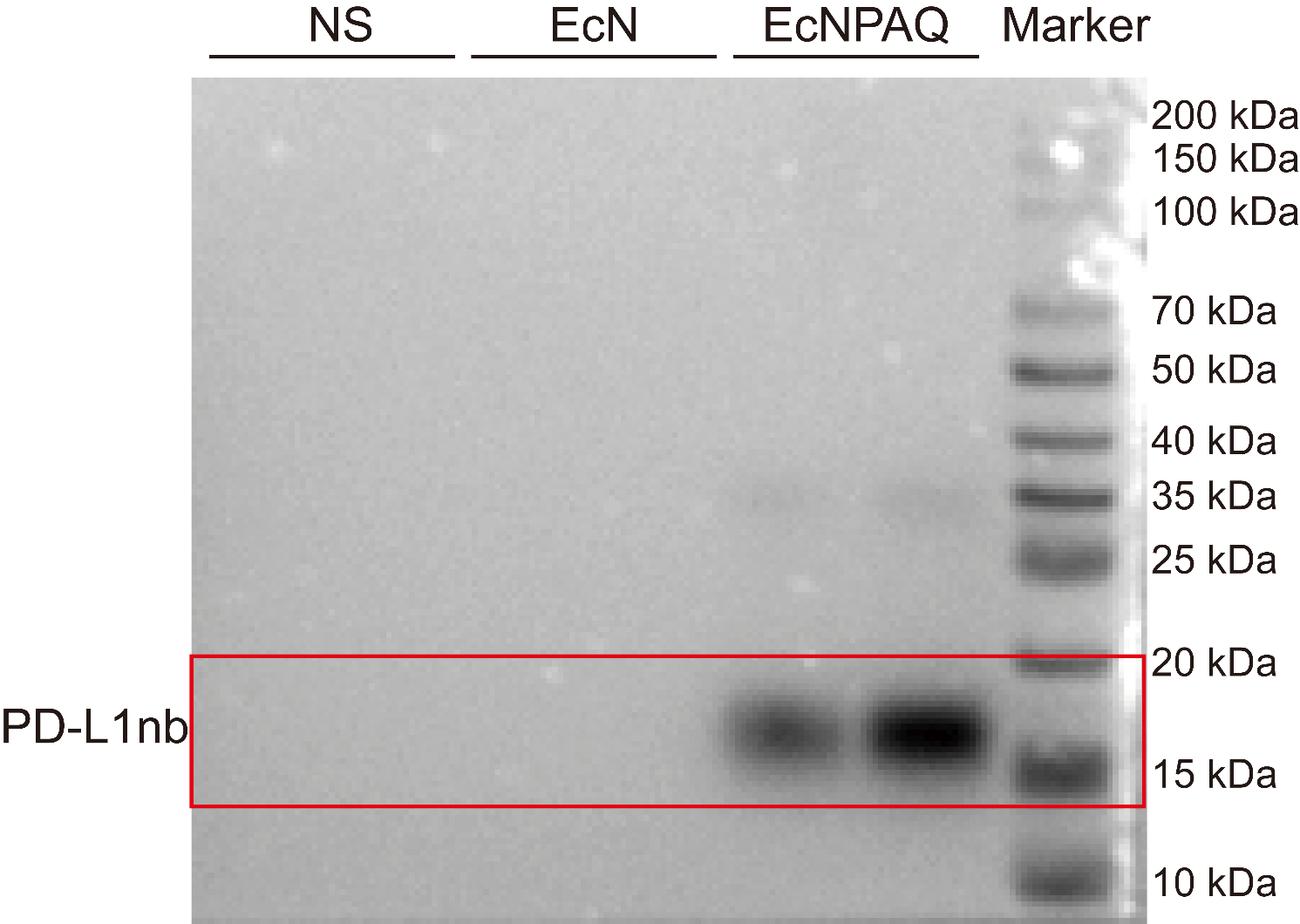


**Figure S5.** Expression of His-tag PD-L1nb across different groups in hPD-L1 MC38 tumor in humanized PD-1 mouse model

Figure S6


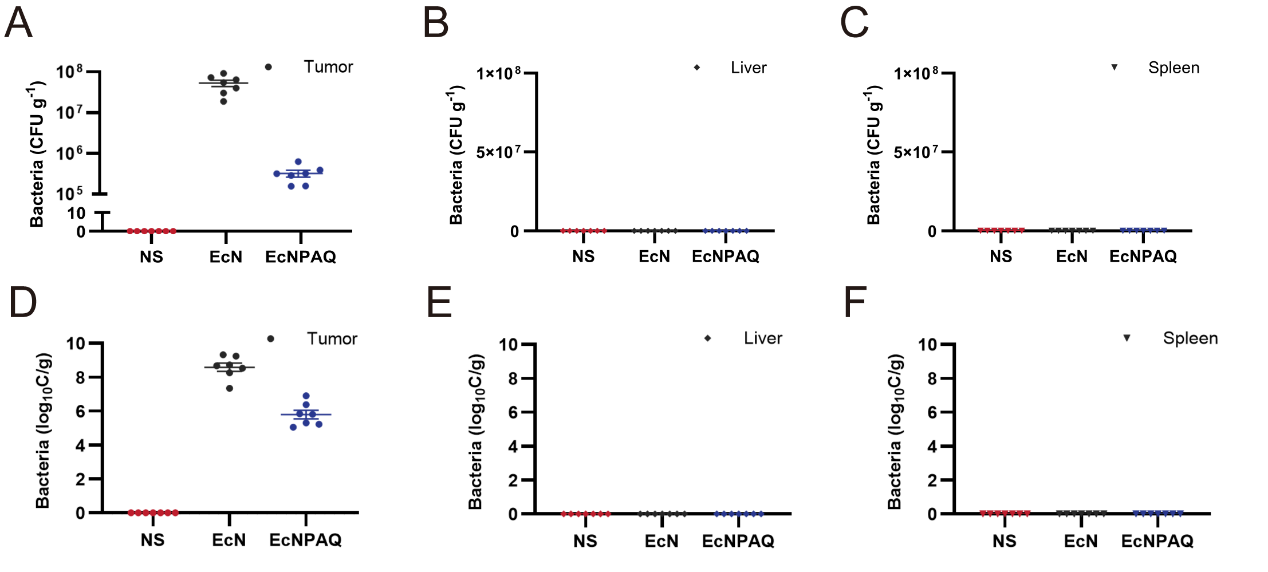


**Figure S6.** In humanized PD-1 mouse model, the biodistribution after intratumoral injection of bacteria. Excised tumors, livers and spleens were homogenized. (A-C) They were plated on LB–agar plates. Colonies were counted to determine CFU/g of tissue. (D-F) They were quantified by qRT-PCR. C is the copy number and EcN were counted as copies/g.

Figure S7


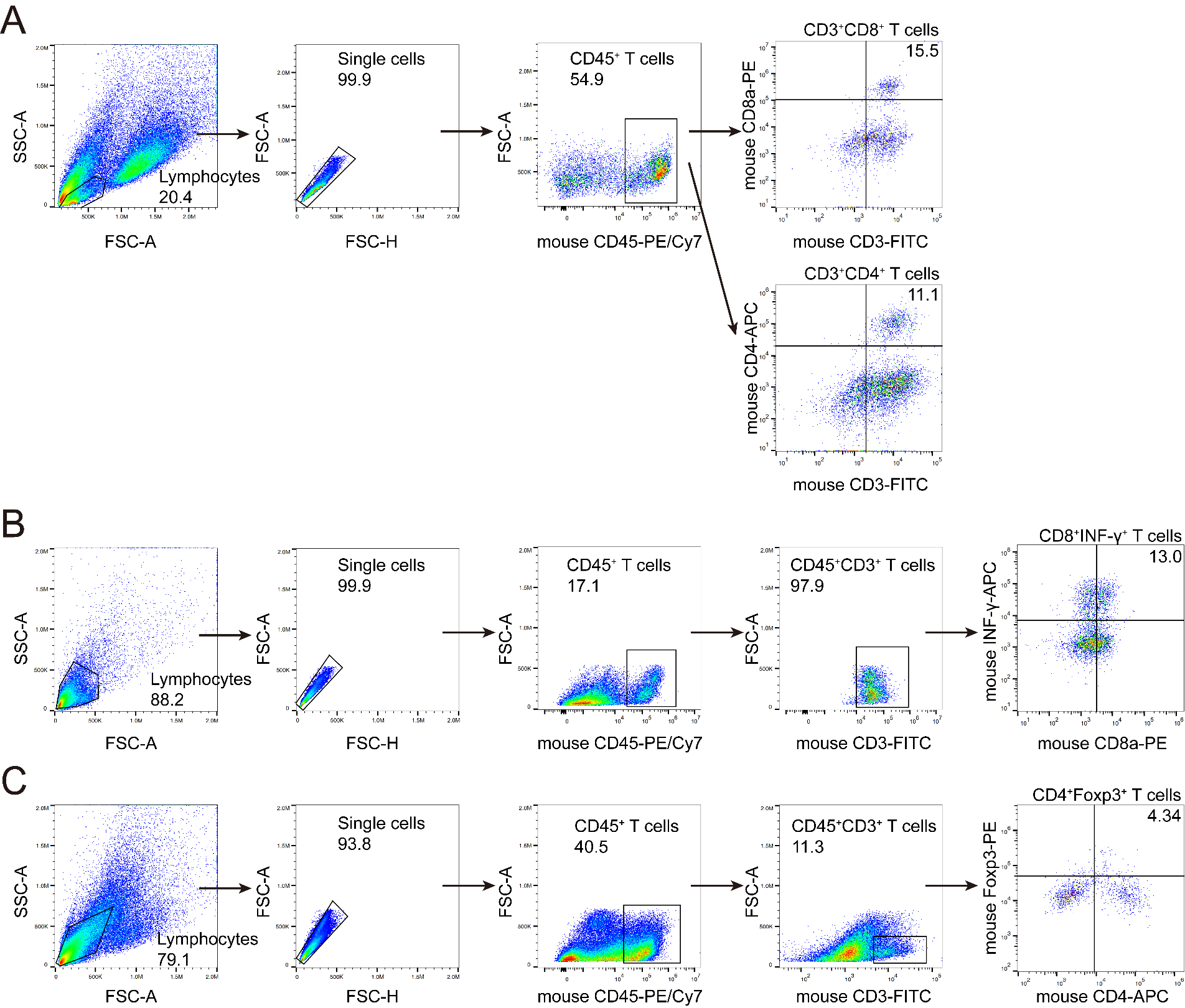


**Figure S7.** In humanized PD-1 mouse model, the gating strategy of mouse CD3^+^CD8^+^ T cells, mouse CD3^+^CD4^+^ T cells (A), mouse CD8^+^INF-γ^+^ T cells (B), and mouse CD4^+^Foxp3^+^ T cells (C).

Figure S8


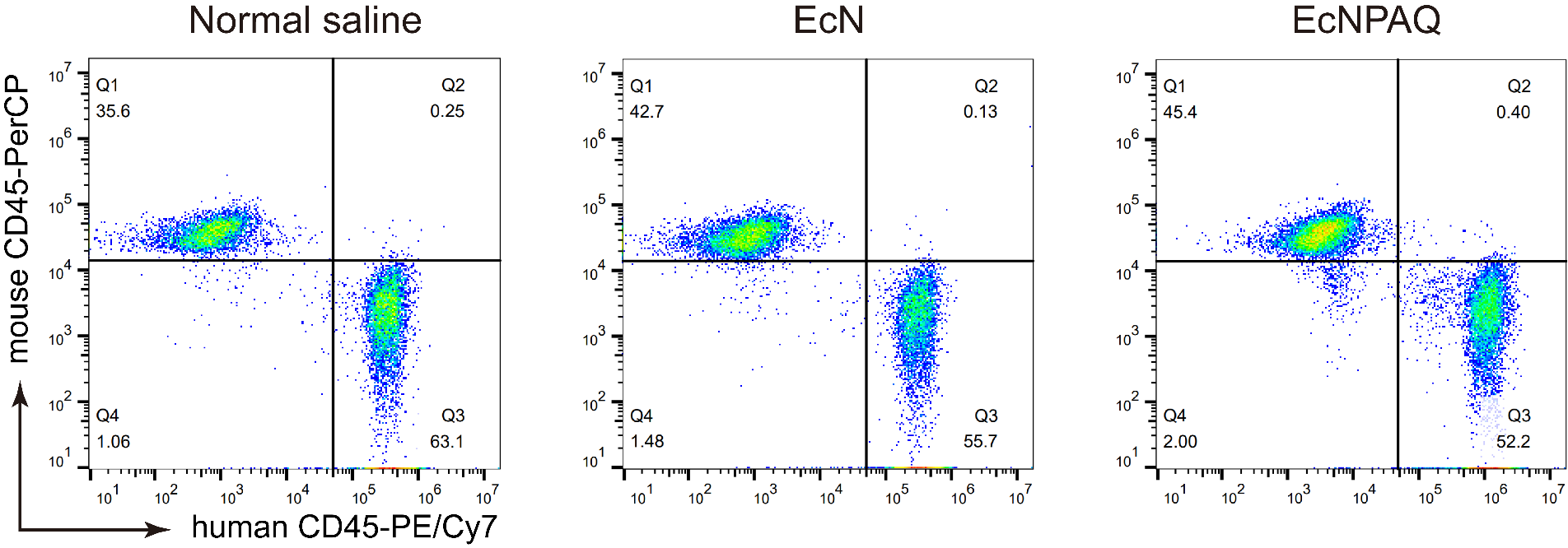


**Figure S8.** Proportion of mCD45^+^ cells and hCD45^+^ cells from three groups on day 14.

Figure S9


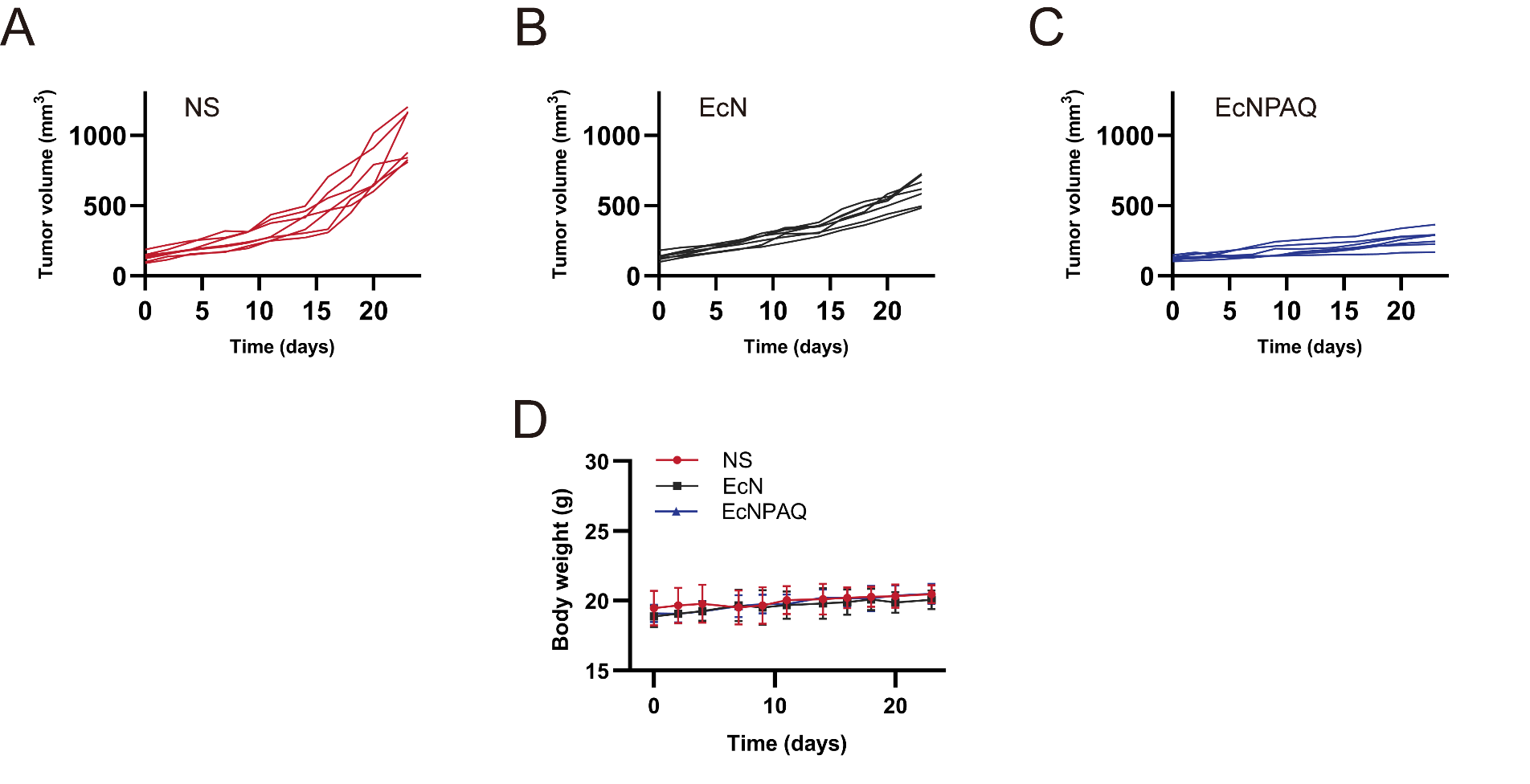


**Figure S9.** In humanized PBMC mouse model, the tumor growth curves of individual mice and the body weight of three groups. n=7.

Figure S10


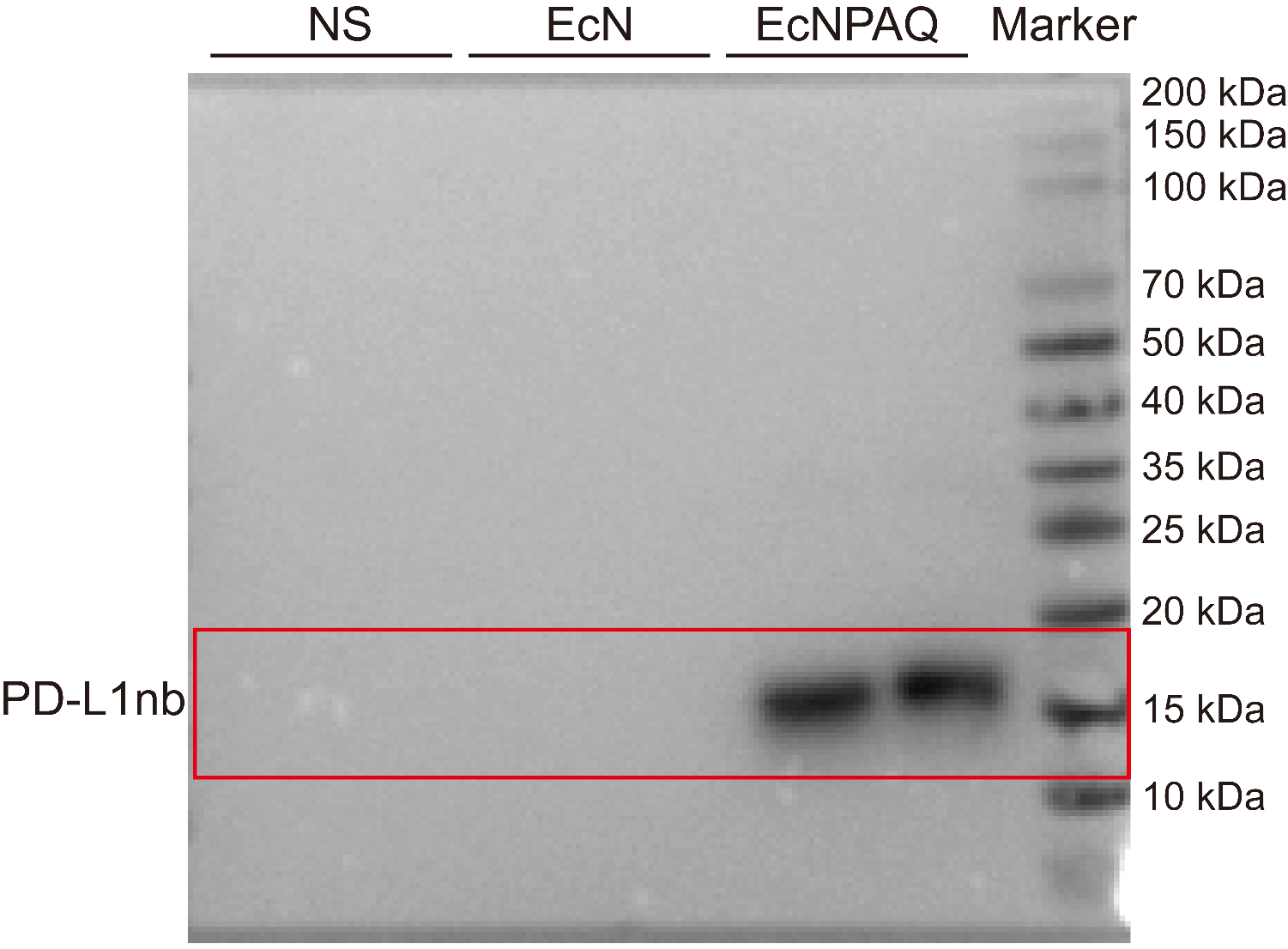


**Figure S10.** Expression of His-tag PD-L1nb across different groups in HT-29 tumor in humanized PBMC mouse model.

Figure S11


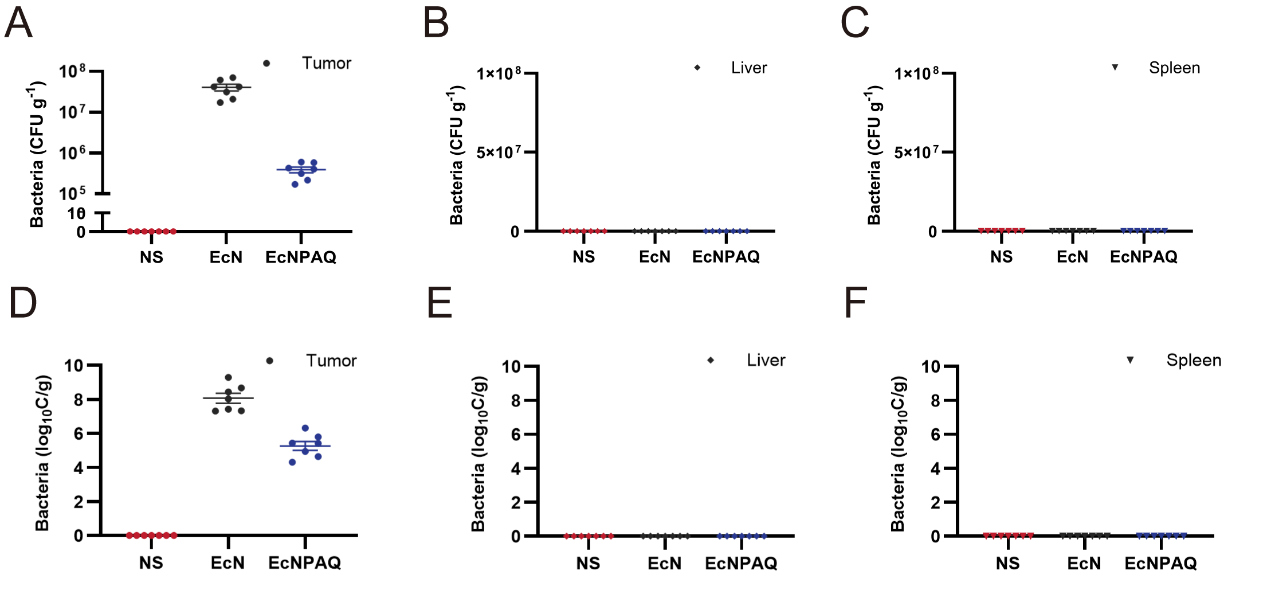


**Figure S11.** In humanized PBMC mouse model, the biodistribution after intratumoral injection of bacteria. Excised tumors, livers and spleens were homogenized. (A-C) They were plated on LB–agar plates. Colonies were counted to determine CFU/g of tissue. (D-F) They were quantified by qRT-PCR. C is the copy number and EcN were counted as copies/g.

Figure S12


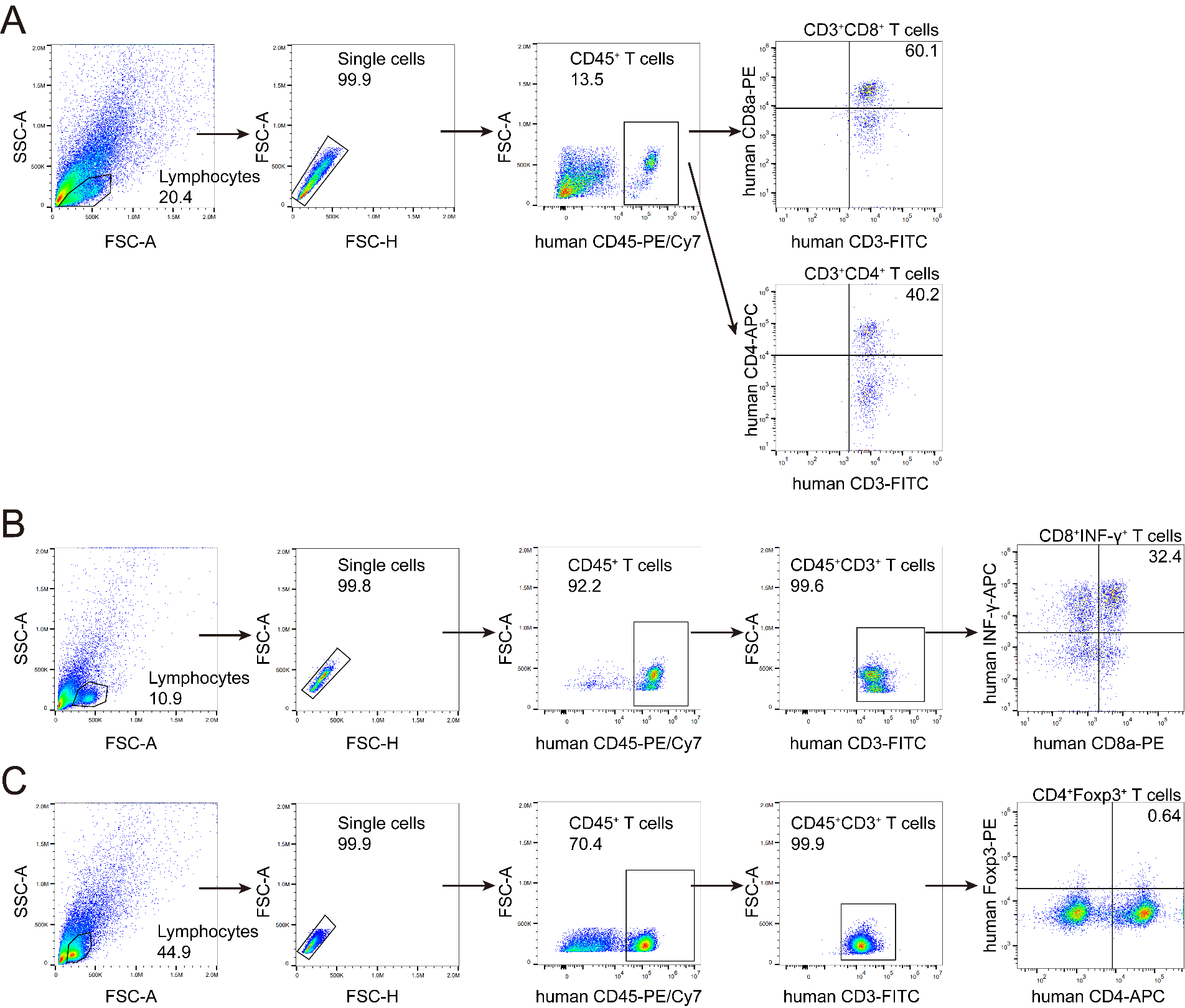


**Figure S12.** In humanized PD-1 mouse model, the gating strategy of human CD3^+^CD8^+^ T cells, human CD3^+^CD4^+^ T cells (A), human CD8^+^INF-γ^+^ T cells (B), and human CD4^+^Foxp3^+^ T cells (C).

Table S1

Strains are used in this article

| Strains | Relevant properties | Sources |
| --- | --- | --- |
| *E. coli* DH5α | F^–^ φ80*lac*ZΔM15 Δ(*lac*ZYA-*arg*F)U169 *rec*A1 *end*A1 *hsd*R17(r_K_^–^, m_K_^+^) *pho*A *sup*E44 λ^–^*thi*-1 *gyr*A96 *rel*A1 | Transgen Biotech |
| *E. coli* BL21(DE3) | F^–^*omp*T *hsd*S_B_ (r_B_^–^, m_B_^–^) *gal dcm*(DE3) | Transgen Biotech |
| *E. coli* Nissle1917 | wild type | Lab stock |
| EcN-FNRS | *E. coli* Nissle1917 harboring pFNRS-*sfGFP* | This study |
| EcN-FF+20 | *E. coli* Nissle1917 harboring pFF+20-*sfGFP* | This study |
| EcN-PpepT | *E. coli* Nissle1917 harboring pPepT-*sfGFP* | This study |
| EcN-pVgb | *E. coli* Nissle1917 harboring pVgb-*sfGFP* | This study |
| EcN-ccdAB | *E. coli* Nissle1917 harboring pJ23114-*ccdB*-pBAD-*ccdA* | This study |
| EcN-CadC | *E. coli* Nissle1917 harboring pCadC-*sfGFP* | This study |
| EcN-LldR | *E. coli* Nissle1917 harboring pLldR-*sfGFP* | This study |
| EcN-pLas-LasR | *E. coli* Nissle1917 harboring pLas-*sfGFP*-J23119-*LasR* | This study |
| EcN-pLas-LuxR | *E. coli* Nissle1917 harboring pLas-*sfGFP*-J23119-*LuxR* | This study |
| EcN-pLas-RpaR | *E. coli* Nissle1917 harboring pLas-*sfGFP*-J23119-*RpaR* | This study |
| EcN-pLas-RhlR | *E. coli* Nissle1917 harboring pLas-*sfGFP*-J23119-*RhlR* | This study |
| EcN-pTra-TraR | *E. coli* Nissle1917 harboring pTra-*sfGFP*-J23119-*TraR* | This study |
| EcN-LuxI | *E. coli* Nissle1917 harboring pTetR-*tetA*-*LuxI* | This study |
| EcN-TraI | *E. coli* Nissle1917 harboring pTetR-*tetA*-*TraI* | This study |
| EcN1 | *E. coli* Nissle1917 knockout *lldD* | This study |
| EcN2 | *E. coli* Nissle1917 knockout *ldhA* | This study |
| EcN3 | *E. coli* Nissle1917 knockout *lldD* and *ldhA* | This study |
| EcN1-ldhA | *E. coli* Nissle1917 knockout *lldD*, harboring pJ23119-*ldhA* | This study |
| EcN2-lldD | *E. coli* Nissle1917 knockout *ldhA*, harboring pJ23119-*lldD* | This study |

| Strains | Relevant properties | Sources |
| --- | --- | --- |
| EcN-ldhA-lldD | *E. coli* Nissle1917 harboring pJ23119-*lldD*-pJ23119-*ldhA* | This study |
| EcNLA | *E. coli* Nissle1917 harboring pJ23119-*LldR*-pLldR-*ldhA*-pJ23114-*TraR*-*LuxI*-*sfGFP* and pCadC-*ccdB*-pTra-*ccdA* | This study |
| EcNPD | *E. coli* Nissle1917 harboring pJ23114-*TraI*-*LasR*-*mRFP* and pLux-*ccdB*-pFF+20-*PD-L1nanobody* | This study |
